# Chemical Variability of Recycled PET Bottles Driven by Packaging Inputs and Processing History: A Marker-Based Multivariate Approach

**DOI:** 10.3390/polym18182194

**Published:** 2026-09-08

**Authors:** Helena Raclavská, Marek Kucbel, Jana Růžičková, Barbora Švédová, Pavel Kantor, Karolina Slamová, Fabrizio Scala

**Affiliations:** 1 ENET Centre, Centre for Energy and Environmental Technologies, VSB–Technical University of Ostrava, 17. Listopadu 15/2172, 708 00 Ostrava, Czech Republic; helena.raclavska@vsb.cz (H.R.); jana.ruzickova@vsb.cz (J.R.); barbora.svedova@vsb.cz (B.Š.); pavel.kantor@vsb.cz (P.K.); 2 Institute of Foreign Languages, VSB–Technical University of Ostrava, 17. Listopadu 15/2172, 708 00 Ostrava, Czech Republic; karolina.slamova@vsb.cz; 3 Department of Chemical, Materials and Industrial Production Engineering, University of Naples Federico II, 80125 Naples, Italy; fabrizio.scala@unina.it; 4 Department of Electronics, Faculty of Electrical Engineering and Computer Science, VSB-Technical University of Ostrava, 17. Listopadu 15/2172, 708 00 Ostrava, Czech Republic

**Keywords:** PET bottles, recycled PET (rPET), NIAS, food contact materials, packaging contamination

## Abstract

Recycled polyethylene terephthalate (rPET) intended for food-contact applications contains complex mixtures of non-intentionally added substances (NIAS) originating from polymer degradation, recycling, packaging-related inputs, and external contamination. Although non-targeted analytical techniques can characterise these complex chemical fingerprints, a standardised framework for their systematic interpretation is lacking. This study introduces a literature-informed, pattern-based framework for interpreting Py-GC/MS fingerprints of commercial PET bottles. Twenty-two commercial PET beverage bottles were analysed by pyrolysis–gas chromatography–mass spectrometry (Py-GC/MS), and detected compounds were organised into four diagnostic profile groups: PET transformation products (C-PET), packaging-associated compounds (E-PS/PVC/FCM), mixed-origin compounds (M-MIX), and condensed aromatic structures (A-AROM). Principal component analysis and a composite Quality Index (QI) were used to evaluate chemical variability. PET transformation products formed a consistent chemical baseline across all samples, whereas chemical variability was primarily associated with packaging-associated compounds and condensed aromatic structures. Bottles containing 100% rPET spanned the full range of chemical profiles, suggesting that the observed variability may be influenced more strongly by feedstock quality, packaging-related inputs, and processing history than by declared recycled content alone. The proposed framework enables a systematic interpretation of Py-GC/MS fingerprints through diagnostic chemical patterns rather than unique source attribution of individual compounds. The proposed framework provides a scalable methodology for comparative evaluation of recycled PET. It highlights that improving chemical quality requires controlling feedstock purity, packaging-related contamination, and processing conditions rather than relying solely on recycled content.

## 1. Introduction

The increasing production and use of plastics have intensified the need for effective recycling strategies, particularly for widely used polymers such as polyethylene terephthalate (PET) [1,2]. Within this context, recycled PET (rPET), especially for food-contact applications, has become a major focus of research and technological development. Although rPET offers clear environmental benefits, its chemical composition is inherently complex and variable because repeated thermal processing during mechanical recycling promotes polymer degradation and the formation of transformation products [3,4].

A key challenge in assessing rPET quality is the presence of non-intentionally added substances (NIAS), which comprise a diverse group of compounds originating from polymer degradation, impurities, and external contamination sources. This complexity has been widely documented in polymer systems [5,6]. In PET systems, NIAS may arise not only from intrinsic degradation pathways but also from external inputs such as inks, adhesives, coatings, and other packaging materials [7,8].

Migration studies have demonstrated that PET-related compounds can transfer into food simulants, highlighting their relevance for material performance and potential consumer exposure [9,10]. Previous studies have further shown that contamination levels in post-consumer PET depend strongly on collection systems, previous use, and mixed input streams, all of which contribute substantially to the chemical heterogeneity of recycled PET [11,12].

This increasing chemical complexity has gained additional regulatory importance following the updated EFSA Scientific Guidance for the safety assessment of post-consumer mechanical PET recycling processes intended for food-contact applications. The guidance, developed in the context of Regulation (EU) 2022/1616 [13], places greater emphasis on feedstock quality, decontamination efficiency, and a comprehensive understanding of non-intentionally added substances throughout the recycling process. Consequently, robust analytical approaches that can systematically interpret complex chemical fingerprints are becoming increasingly important not only for material development but also for regulatory evaluation of recycled PET intended for food-contact applications [14].

Despite extensive research on PET degradation, recycling technologies, and contamination pathways, current analytical approaches remain focused primarily on compound-specific identification, migration studies, or process-level evaluation. Recent investigations increasingly recognise that individual NIAS may originate from multiple concurrent pathways, including polymer degradation, thermal reprocessing, additives, packaging components, collection systems, and cross-contamination, making definitive source attribution of individual compounds inherently difficult [8,15]. At the same time, non-targeted screening combined with chemometric analysis has shifted the emphasis from identifying individual compounds to interpreting complete chemical fingerprints [15,16]. However, despite these advances, no standardised framework is currently available for systematic interpretation and comparison of Py-GC/MS fingerprints across heterogeneous commercial PET materials.

Non-targeted analytical techniques such as pyrolysis–gas chromatography–mass spectrometry (Py-GC/MS) have therefore gained increasing attention for polymer characterisation [17,18]. Py-GC/MS generates transformation-derived chemical fingerprints that reflect the cumulative effects of polymer degradation, processing history, recycling, additives, packaging-related inputs, and contamination, rather than the native composition of the material itself [19,20]. Consequently, interpretation increasingly relies on recognising patterns within complete chemical fingerprints rather than assigning unique origins to individual compounds [8,16].

**Figure 1:**
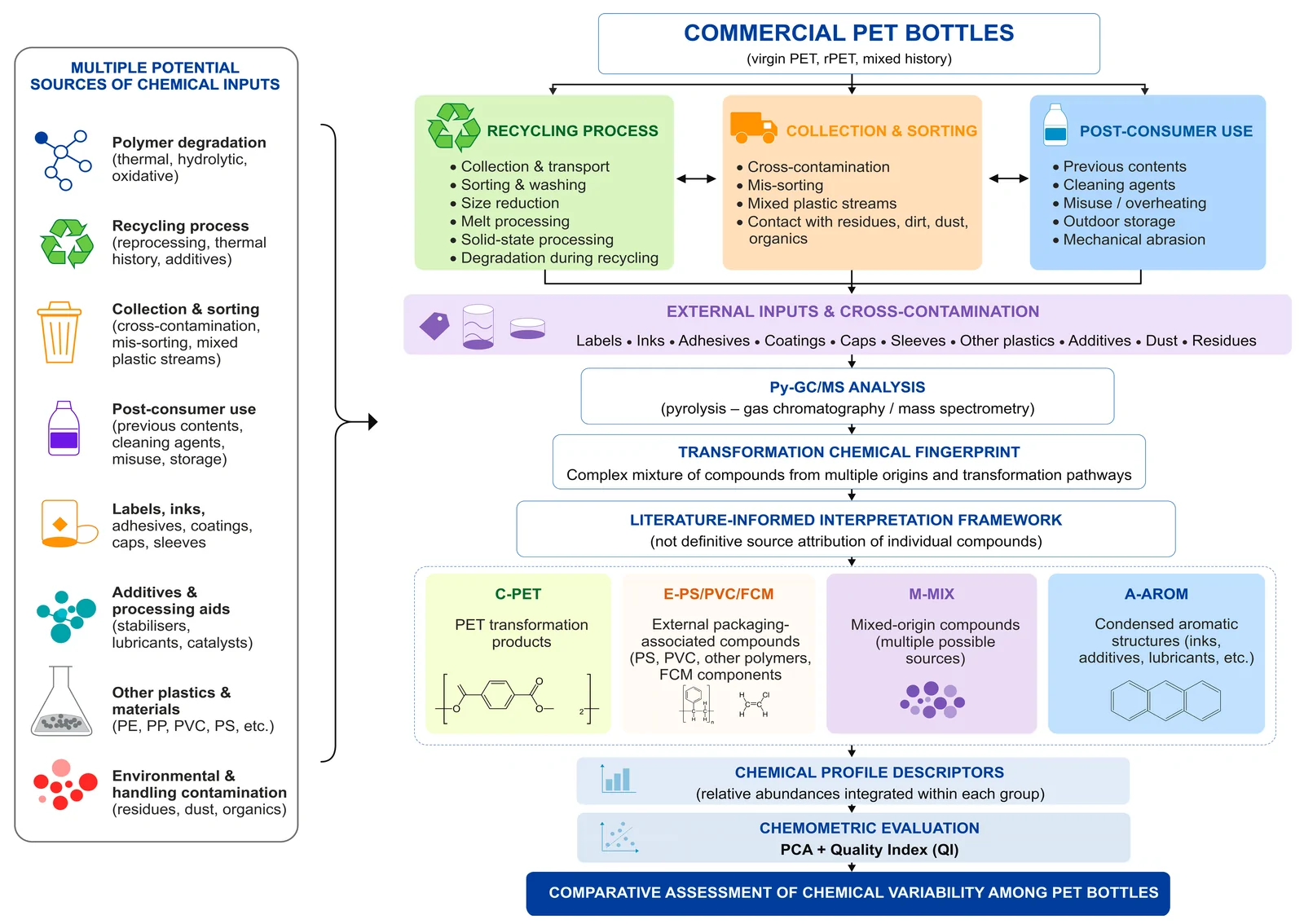
Conceptual framework of the proposed literature-informed interpretation of Py-GC/MS fingerprints in commercial PET bottles.

To address this gap, the present study introduces a literature-informed, pattern-based interpretation framework for Py-GC/MS fingerprints of commercial PET bottles (Figure 1). Instead of treating individual compounds as unique source-specific markers, the proposed framework organises compounds into diagnostic profile groups according to their predominant literature associations and interprets the resulting chemical fingerprints using complementary chemometric tools, including principal component analysis (PCA) and a composite Quality Index (QI). The conceptual workflow of the proposed framework is summarised in Figure 1.

To the best of our knowledge, this study introduces the first literature-informed framework for systematic interpretation of Py-GC/MS fingerprints of commercial PET bottles. Rather than proposing new marker compounds, the framework integrates diagnostic profile groups, multivariate chemometric analysis, and a composite Quality Index into a unified framework for comparative evaluation of chemical variability. By explicitly acknowledging the multifactorial origin of many NIAS, the proposed framework provides a scalable analytical methodology for material comparison, quality assessment, and future analytical evaluation of recycled food-contact PET materials.

## 2. Materials and Methods

### 2.1. Materials

Twenty-two post-consumer PET beverage bottles were collected from the Czech retail market to represent commonly used packaging formats for soft drinks and bottled water; twenty were selected for chemical analysis. The sample set included bottles of beverages from international beverage groups, such as The Coca-Cola Company (Atlanta, GA, USA), as well as regional beverage companies, including Kofola ČeskoSlovensko a.s. (Ostrava, Czech Republic) and Mattoni 1873 a.s. (Karlovy Vary, Czech Republic).

According to publicly available information, some bottles were declared to contain 100% recycled PET (rPET), while others were produced from blends of virgin and recycled PET. These declarations were used solely for classification purposes and were not independently verified. An overview of bottle characteristics, beverage types, and rPET declarations is provided in Table 1, with further details given in Table S1.

### 2.2. Methods

#### 2.2.1. Py-GC/MS Analysis

PET bottles were size-reduced using a Retsch SM-series cutting mill and subsequently cryogenically milled under liquid nitrogen (RETSCH GmbH, Haan, Germany) to obtain particles below 0.5 mm. Subsamples of 100–500 µg were analysed in duplicate, and reported values represent arithmetic means. Replicate variability was low, consistent with the reproducibility of single-shot Py-GC/MS for polymer fingerprinting.

Pyrolysis was performed using a Frontier Lab AS2020E autosampler (Frontier Laboratories Ltd., Koriyama, Japan) coupled to a microfurnace pyrolyser and connected to an Agilent 7890 GC/5977C MSD system (Agilent Technologies, Inc., Santa Clara, CA, USA). Samples were heated at 600 °C for 60 s under a single-shot program. These conditions were selected to ensure reproducible and extensive thermal decomposition of PET, consistent with established analytical pyrolysis methodologies [20,21]. The selected temperature and operational parameters are also consistent with more recent studies applying single-shot Py-GC/MS for polymer characterisation and chemical fingerprinting under comparable analytical conditions [22,23]. Pyrolysates were transferred into the GC inlet at 300 °C using pulsed split injection (1:500). Separation was achieved on an Ultra ALLOY^®^ UA+Py2 (Frontier Laboratories Ltd., Koriyama, Japan) metal capillary column (30 m × 0.25 mm × 0.50 µm) using the following oven program: 40 °C (2 min), ramped to 320 °C at 20 °C min^−1^ (2 min hold), followed by a second ramp from 280 °C to 320 °C at 30 °C min^−1^ (5 min hold). Mass spectra were acquired in full-scan mode. Compound identification was performed using the F-Search software (v3.8; Frontier Laboratories Ltd., Koriyama, Japan) with a library of polymer-specific pyrolysis spectra. All identifications were considered tentative because Py-GC/MS generates transformation products rather than directly detecting the native chemical composition of PET materials. Consequently, individual compounds were not interpreted as unique source-specific markers.

The overall interpretation strategy adopted in this study is illustrated in Figure 1. Py-GC/MS was applied as a fingerprint-generating technique, producing transformation-derived chemical profiles that reflect the combined effects of polymer degradation, thermal processing, recycling history, additives, packaging-related inputs, and potential cross-contamination. Because individual compounds may originate from multiple concurrent pathways, interpretation was performed at the level of diagnostic profile groups representing predominant literature associations rather than definitive source attribution. The resulting chemical descriptors were subsequently integrated by multivariate analysis (PCA) and the composite Quality Index (QI) to enable comparative evaluation of chemical variability among commercial PET bottles.

Py-GC/MS generates transformation-derived chemical fingerprints reflecting the combined effects of polymer degradation, recycling, packaging-related inputs, previous use, and cross-contamination. Rather than assigning unique sources to individual compounds, the proposed framework interprets chemical fingerprints using diagnostic profile groups representing predominant literature associations. These descriptors are then integrated using principal component analysis (PCA) and the composite Quality Index (QI) to compare chemical variability among PET bottles.

#### 2.2.2. Calculation of PET Mass per Litre

Twenty-two PET bottles (0.5–1.5 L) were weighed using a Sartorius A200S balance (Sartorius GmbH, Göttingen, Germany) excluding the bottle caps. Recorded parameters included bottle mass, cap mass, nominal volume, bottle colour, manufacturer, and declared rPET content (Table 1). PET mass per litre was calculated as in Equation (1):

(1) $$\mathrm{PET}\;\mathrm{mass}\;\mathrm{per}\;\mathrm{litre}\;(g/L)=\frac{\mathrm{bottle}\;\mathrm{mass}\;\left(g\right)}{\mathrm{nominal}\;\mathrm{volume}\;\left(L\right)}$$

This normalisation accounts for fixed structural components of the bottles (neck, thread, and base) that prevent linear scaling of mass with volume, as noted in previous packaging life-cycle assessment studies [24,25]. No surface-area corrections were applied.

#### 2.2.3. Statistical Analysis and Marker-Based Evaluation

Relative abundances of detected compounds were expressed as percentage peak areas and used for semi-quantitative comparison and multivariate analysis. Principal component analysis (PCA) was performed in the Python (v3.13) software environment using the pandas (v2.3), NumPy (v2.1), scikit-learn (v1.7), and Matplotlib (v3.9) libraries. (Python Software Foundation, Wilmington, DE, USA). Aggregated data for the four diagnostic profile groups defined in Section 3.2 were used: PET transformation products (C-PET), packaging-associated compounds (E-PS/PVC/FCM), mixed-origin compounds (M-MIX), and condensed aromatic structures (A-AROM). These groups represent predominant literature associations within Py-GC/MS transformation fingerprints rather than definitive source attribution of individual compounds.

PCA was conducted on the total relative contributions per group. The data matrix was mean-centred and scaled to unit variance to ensure that differences in magnitude between marker groups did not bias the PCA results, following standard chemometric practice [26]. PC1 and PC2 were retained for interpretation. PC1 represented the dominant axis of chemical variability and was primarily associated with overall differences in diagnostic profile composition, particularly contributions of C-PET, E-PS/PVC/FCM, and A-AROM. PC2 captured secondary variability reflecting differences in the relative contributions of individual marker groups, particularly A-AROM and M-MIX. The resulting components enabled comparative evaluation of chemical heterogeneity across samples [27,28].

##### Identification of Variables Contributing to PCA Separation

To aid interpretation, an additional PCA model was performed at the compound level. Compounds with the highest absolute loadings on PC1 and PC2 were visualised in a loading plot. Loading plots allow identification of representative markers that show sample differentiation by showing the contribution of individual compounds to each principal component. This complements the primary PCA on aggregated marker groups, facilitating a detailed understanding of the chemical drivers underlying sample variability.

#### 2.2.4. Pattern-Based Interpretation Framework

The proposed framework organises compounds into four diagnostic profile groups (C-PET, E-PS/PVC/FCM, M-MIX, and A-AROM) to enable systematic interpretation of Py-GC/MS fingerprints obtained from commercial PET bottles (Figure 1). Assignment of compounds to individual groups was based on their predominant literature associations and their relevance for interpretation of transformation-derived chemical fingerprints rather than on exclusive source attribution.

Py-GC/MS generates transformation products that may originate from multiple concurrent pathways, including polymer degradation, thermal reprocessing, additives, packaging components, previous use, collection systems, and cross-contamination. Consequently, individual compounds were not interpreted as unique source-specific markers. Instead, the proposed framework evaluates the combined occurrence of compounds within diagnostic profile groups representing the dominant chemical processes contributing to the overall fingerprint.

For comparative evaluation, samples were categorised into low, medium, and high levels of diagnostic profile intensity based on empirical percentile thresholds. These categories provide a relative measure of chemical variability among PET bottles and are intended solely for comparative interpretation. They do not represent toxicological classification or forensic source identification.

The framework provides a structured, reproducible approach to integrating Py-GC/MS fingerprints with multivariate analysis (PCA) and the composite Quality Index (QI), enabling systematic comparison of chemical variability among commercial PET bottles.

#### 2.2.5. Composite Quality Index

A Composite Quality Index (QI) was developed to summarise chemical patterns across samples (Equation (2)):

(2) $$QI_{i}=\frac{1}{\left(E_{i}+M_{i}+{2A}_{i}+\varepsilon \right)}$$

where *E_i_*, *M_i_* and *A_i_* represent the relative contributions of the E-PS/PVC/FCM, M-MIX, and A-AROM diagnostic profile groups, respectively, and is a small constant introduced to avoid division by zero; ε is a small positive constant introduced to avoid division by zero.

The weighting of A-AROM reflects its disproportionately strong contribution to chemical variability observed in the dataset. This contribution was particularly evident along PC1, where A-AROM contributed substantially to sample differentiation together with the packaging-associated diagnostic profile group (E-PS/PVC/FCM). The applied weighting does not represent a mathematically optimised coefficient but rather a pragmatic scaling factor selected empirically based on the observed multivariate structure of the dataset. Its purpose is to ensure that the Quality Index adequately reflects the relative influence of condensed aromatic structures while maintaining a simple, transparent, and readily interpretable formulation.

The index is intended as a comparative descriptor rather than a toxicological metric, following established principles for composite indicators [29] and recent approaches in recycled polymer assessment [27,30]. The applied weighting increases the influence of aromatic transformation behaviour on the final QI but does not alter the overall interpretation of the multivariate patterns, which remains consistent with the PCA results. QI values were evaluated in relation to PCA to assess whether the index reflects dominant variance in marker-group distributions.

#### 2.2.6. Data Processing/Compound Selection

A prevalence-based filtering approach was applied to define a core set of consistently occurring compounds across samples. Compounds detected in at least 11 of 20 bottles (≥55%) were retained for further evaluation. The threshold was selected empirically to balance representativeness and robustness of the dataset by retaining compounds occurring consistently across the analysed commercial PET bottles while excluding sporadic or sample-specific compounds that would reduce the robustness of comparative interpretation. A higher threshold would have excluded several diagnostically informative compounds. In contrast, a substantially lower threshold would have retained numerous low-frequency compounds with limited comparative value, consistent with prevalence-based filtering approaches used in the analysis of complex chemical datasets [31]. The resulting core dataset served as the basis for assignment to the diagnostic profile groups, and all quantitative evaluations presented in Tables S3–S5 were calculated exclusively from this filtered dataset.

## 3. Results

### 3.1. PET Mass per Litre as a Descriptor of Material Intensity

**Figure 2:**
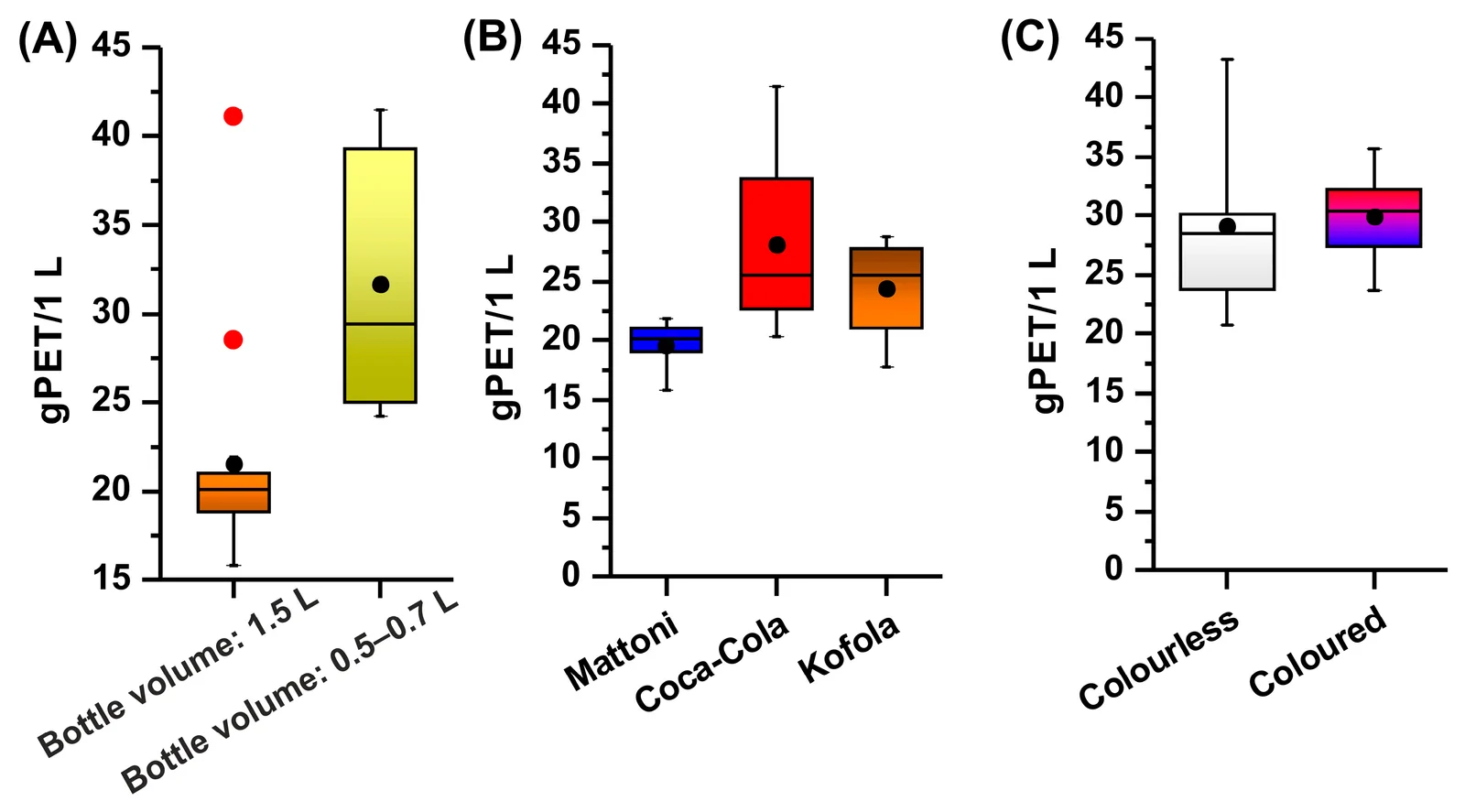
Boxplot (**A**) PET mass per litre across two volume categories (1.5 L vs. 0.5–0.7 L), red dots indicate outliers; (**B**) manufacturer-specific PET usage in 1.5 L bottles; (**C**) PET usage in colourless vs. coloured bottles.

PET mass per litre provides a standardised measure of material use, enabling direct comparison across bottle sizes and designs. It expresses the amount of plastic relative to beverage volume and is commonly used as an indicator of material intensity in packaging systems [24,25]. For 1.5 L bottles, values ranged from approximately 15.8 to 22.0 g/L, whereas smaller 0.5–0.7 L bottles exhibited higher values of 25–40 g/L, with some outliers exceeding 41 g/L (Figure 2A). This reflects the disproportionate contribution of fixed structural components, such as the neck and base, in smaller bottles, supporting the use of g/L normalisation for cross-volume comparison.

Individual measurements for 1.5 L bottles (Figure 2B) showed a wider range (~16–42 g/L). Mattoni bottles consistently showed lower PET mass with limited variability; Coca-Cola bottles were higher and more variable; and Kofola bottles were intermediate. Comparison of colourless and coloured bottles (Figure 2C) showed overlapping ranges, with slightly higher median PET mass in coloured bottles (~30 g/L) than in clear bottles (~28–29 g/L), likely reflecting design choices rather than material colour.

No systematic differences were observed between bottles declared as 100% rPET and those containing partial recycled content (e.g., 25–35%). For 1.5 L bottles, 100% rPET ranged from approximately 18–22 g/L, while partially recycled PET ranged from 17 to 29 g/L. These findings indicate that material use is primarily determined by bottle design and manufacturing specifications rather than recycled content. In summary, PET mass per litre represents a practical descriptor of material intensity and design optimisation. Its relationship to chemical profile variability is evaluated in the following sections.

### 3.2. Pattern-Based Interpretation of Py-GC/MS Fingerprints

Py-GC/MS analysis generated complex chromatographic profiles for all analysed PET bottle samples, reflecting the thermal decomposition behaviour of the material. The detected compounds comprised a wide range of low-molecular-weight species, including fragments associated with PET backbone degradation, as well as compounds linked to external materials and mixed-origin structures. Although individual compounds were tentatively identified using spectral libraries, the complexity of the profiles and the nature of pyrolysis reactions limit direct compound-level interpretation. Therefore, following the literature-informed framework described in Section 2.2.4, subsequent analysis focused on aggregated chemical patterns represented by diagnostic profile groups rather than on definitive source attribution of individual compounds.

Detected compounds were assigned to four diagnostic profile groups: (i) PET transformation products (C-PET); (ii) packaging-associated compounds (E-PS/PVC/FCM); (iii) mixed-origin compounds (M-MIX); and (iv) condensed aromatic structures (A-AROM).

The C-PET group formed a consistent background across all samples, reflecting common PET transformation pathways, with variability primarily quantitative rather than compositional.

In contrast, the E-PS/PVC/FCM group showed substantial variability between samples, indicating differences in packaging-related inputs such as labels, adhesives, printing inks, and other packaging-related materials.

The M-MIX group represented compounds with ambiguous or overlapping literature associations, likely resulting from interactions between PET degradation products and external materials during thermal processing. Although present in lower relative abundance, this group contributed to the overall complexity of the chemical profiles.

**Figure 3:**
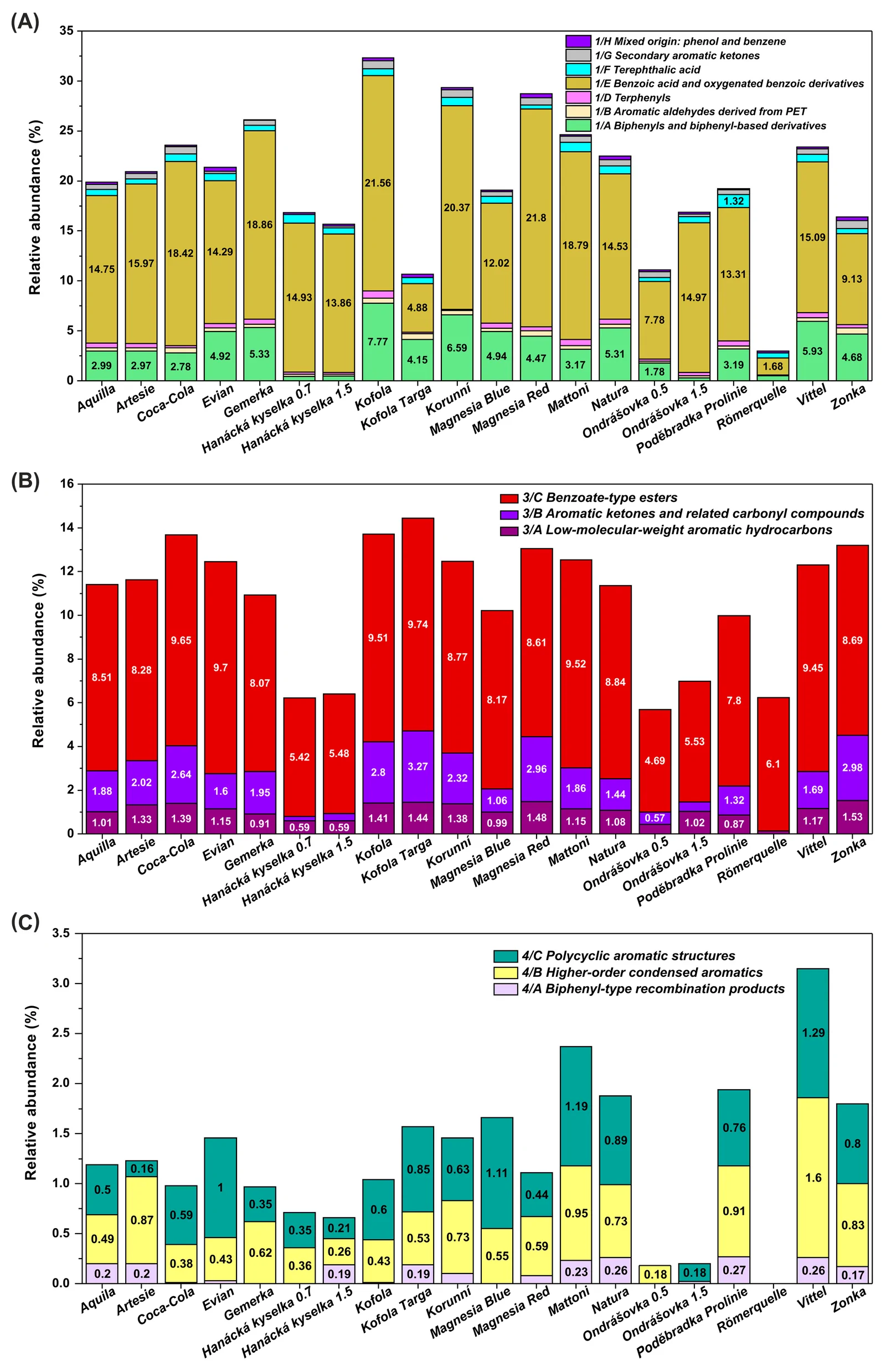
Relative contributions of major marker-group subcategories across PET bottles based on percentage peak areas: (**A**) C-PET subgroup concentrations, including biphenyl derivatives, aromatic aldehydes, terphenyls, and benzoic derivatives; (**B**) E-PS/PVC/FCM subgroup concentrations, including aromatic hydrocarbons, aromatic ketones, and benzoate esters; (**C**) A-AROM subgroup concentrations, including biphenyl-type recombination products, condensed aromatic structures, and polycyclic aromatic structures.

The A-AROM group comprised condensed aromatic structures formed during pyrolysis through recombination and condensation reactions. Relative abundances varied markedly between samples, reflecting differences in aromatic content, contamination pathways, or material history. These compounds are interpreted as transformation-derived features rather than direct indicators of their presence in the original PET material. Detailed quantitative data for the diagnostic profile groups are provided in Tables S3–S5, and Figure 3 illustrates their relative contributions across all analysed PET bottles.

#### 3.2.1. Chemically Transformed PET-Derived Compounds (C-PET)

All analysed bottles exhibited a consistent qualitative C-PET pattern. C-PET compounds represent aromatic transformation products formed during thermal, oxidative, and processing-related degradation of PET through mechanisms including ester bond scission, decarboxylation, oxidation, and radical recombination [20,32]. The consistent occurrence of these compounds across samples supports their assignment to the C-PET diagnostic profile group. However, the present analytical framework cannot directly differentiate virgin from recycled PET. To facilitate structured interpretation, C-PET compounds were assigned to mechanistic subgroups representing distinct transformation pathways within PET degradation chemistry:

- 1/A Biphenyls and derivatives—from aromatic radical recombination following ester bond scission and decarboxylation, further alkylated, vinylated, and oxidised.
- 1/B Aromatic aldehydes—e.g., benzaldehyde and benzeneacetaldehyde—from oxidation of benzoyl-type intermediates [33,34].
- 1/C Diphenylmethane—methylene-bridged aromatic fragments, excluded from the core set due to prevalence < 55%.
- 1/D Terphenyls—higher-order aromatics via secondary aryl–aryl condensation.
- 1/E Benzoic acid and oxygenated derivatives—cumulative oxidative transformation products.
- 1/F Terephthalic acid—marker of polymer chain degradation via hydrolytic and thermo-oxidative cleavage.
- 1/G Secondary aromatic ketones—from secondary acylation/alkylation of PET-derived aromatics.
- 1/H Mixed-origin low-molecular-weight aromatics—e.g., phenol and benzene—may originate from PET degradation or external inputs [7,35].

##### C-PET Quantitative Characteristics

Across all samples, the C-PET profile was qualitatively consistent, with terephthalic acid as the dominant transformation product. Biphenyls, aromatic aldehydes, terphenyls, and low-molecular-weight aromatics formed a characteristic suite of PET-derived compounds. A core set was defined using a prevalence threshold of ≥11 out of 20 bottles (≥55%), reducing the initial set of 20 compounds to 16 core compounds, with Diphenylmethane excluded. This resulted in seven retained structural subgroups for final evaluation (Tables S2 and S3).

Quantitatively, terephthalic acid contributed most to the overall C-PET profile, whereas biphenyls and terphenyls were the primary contributors to inter-sample variability. Benzene and phenol were present at low but relatively stable levels, consistent with their volatility and diffusion behaviour in PET matrices [36,37].

Based on the relative contributions of biphenyls, terphenyls, aromatic aldehydes, and low-molecular-weight aromatics, a gradient of transformation intensity was observed across the analysed bottles. Samples associated with plain waters generally exhibited lower contributions of higher-order aromatics and more stable aldehyde profiles. In contrast, soft drinks typically showed higher relative abundances and variability of these compounds (Table S4). Figure 3A summarises the distribution of major C-PET subgroups across PET bottles.

#### 3.2.2. Compounds of Mixed or Ambiguous Origin (M-MIX)

The M-MIX group comprises compounds with mixed or ambiguous literature associations that cannot be unambiguously attributed to either PET transformation or external packaging-related inputs, representing overlapping transformation pathways and background chemical complexity. Ethylene glycol mono- and dibenzoates were included because their formation is not typical of primary PET degradation and may also reflect contributions from adhesives, coatings, inks, or plasticiser formulations [7,38]. (E)-Stilbene was assigned to M-MIX due to its possible association with both severe thermal transformation and external materials such as labels, inks, or mixed-polymer inputs [39,40].

Across all analysed bottles, the M-MIX group comprised three compounds, of which two recurrent compounds were retained in the core dataset. Quantitatively, M-MIX exhibited relatively low variability across samples, with total contributions generally ranging between approximately 2 and 3%. No consistent trends were observed with respect to beverage type, bottle colour, or transformation-intensity category. The relatively stable distribution indicates that M-MIX compounds represent a background component of chemical complexity rather than primary drivers of inter-sample differentiation. Because of their relatively low variability and limited discriminative value across samples, M-MIX compounds were not visualised separately in Figure 3, which focuses primarily on diagnostic profile groups exhibiting stronger differentiation patterns among PET bottles.

#### 3.2.3. Compounds Associated with External Inputs (E-PS/PVC/FCM)

The E-PS/PVC/FCM group comprises compounds associated with external materials, including polymers, additives, and food-contact-related components, rather than PET transformation processes. This group includes low-molecular-weight aromatics (e.g., BTX-type compounds), styrenic derivatives, aromatic ketones, benzoate esters, and selected plasticiser-related compounds, reflecting the presence of packaging-related inputs such as labels, inks, coatings, adhesives, and mixed-polymer streams [7]. Following the literature-informed framework, compounds were assigned to structural subgroups according to their predominant literature associations and analytical relevance within the overall Py-GC/MS fingerprint:

- 3/A Low-molecular-weight aromatic hydrocarbons—BTX-type compounds, styrene, ethylbenzene, indane, and alkyl-substituted benzenes, commonly associated with styrenic materials and packaging-related inputs.
- 3/B Aromatic ketones and related carbonyl compounds—including acetophenone derivatives, propiophenones, and fluorenone-type structures—are typically linked to photoinitiators and UV-curable coatings [41,42].
- 3/C Benzoate-type esters—aromatic esters such as cyanophenyl benzoates, butylbenzoates, and diethylene glycol dibenzoate, widely used in adhesives, coatings, and printing inks [43].
- 3/D Plasticiser-related compounds—phthalate, terephthalate, and adipate esters (e.g., DEP, DEHA, DEHT, bis(2-methylpropyl)phthalate), representing external ester-based additives [44].
- 3/E Other additive-related markers—low-frequency compounds, including triphenylphosphine oxide.

Plasticiser-related compounds (3/D), although excluded from the core set, are reported here due to their potential regulatory relevance, with concentrations ranging from 0.11% to 9.50% for individual compounds. Samples were grouped by beverage type (plain waters, flavoured mineral waters, and soft drinks) to facilitate comparison. Benzoate-type esters (3/C) were consistently detected and formed the dominant component of the group. Plain waters displayed relatively narrow variability (6.2–12.5% total contribution, 8–21 compounds); flavoured mineral waters showed broader variability (5.7–13.1%; 14–21 compounds); and soft drinks exhibited the highest contributions (13.2–14.5%; 19–22 compounds). Within each beverage category, individual bottles differed in total contributions and compound counts, reflecting packaging complexity and processing history.

In summary, the E-PS/PVC/FCM group represents a major driver of chemical variability across PET bottles. Differences are primarily quantitative, driven by the composition and complexity of packaging-related inputs, while the consistent presence of benzoate esters forms a baseline for comparative interpretation. Subgroups 3/A and 3/B contributed most strongly to inter-sample variability, whereas the lower-prevalence subgroups 3/D and 3/E provide additional analytical context and regulatory relevance. The relative contributions of the E-PS/PVC/FCM subgroup categories are shown in Figure 3B.

##### Plasticiser-Related Compounds (Subgroup 3/D)

Plasticiser-related compounds were detected in a subset of samples, including diethyl phthalate (DEP), DEHA, DEHT, and bis(2-methylpropyl) phthalate. Detected concentrations varied across samples: bis(2-methylpropyl) phthalate ranged from approximately 0.11 to 1.08%, DEP from 0.02 to 9.50%, DEHT from 0.56 to 2.74%, and DEHA from 0.15 to 0.49%. No consistent relationship was observed between concentration levels and beverage type or other grouping variables. Given their limited occurrence frequency and variable distribution, plasticiser-related compounds were not included in the core comparative analysis but are reported here as part of the overall compositional dataset. Their presence nevertheless highlights the relevance of packaging-related additives in shaping the chemical profiles of PET bottles.

#### 3.2.4. Condensed Aromatic Structures (A-AROM)

The A-AROM group comprises condensed aromatic structures detected within the pyrolysis profiles, including compounds structurally related to polycyclic and multi-ring aromatic hydrocarbons. These structures arise predominantly from high-temperature radical recombination, aromatic condensation, and secondary transformation processes occurring during the pyrolysis of aromatic polymer systems such as PET. These processes have been described in previous studies [20,32] and have been observed in recent analyses of recycled PET [45]. Under the applied analytical conditions, aromatic fragments generated from terephthalate units undergo recombination and condensation reactions, leading to increasingly complex aromatic structures. Such transformations are well documented in pyrolysis studies of synthetic polymers and reflect transformation pathways rather than the direct presence of these compounds in the original material [3,46]. Accordingly, the detected A-AROM compounds are interpreted as indicators of transformation behaviour and structural recombination patterns rather than direct evidence of their presence in the analysed PET bottles before pyrolysis. Following the literature-informed framework, detected compounds were assigned to four structural subcategories reflecting increasing degrees of aromatic condensation:

- 4/A Biphenyl-type recombination products (e.g., dimethylbiphenyl, binaphthalene), formed via recombination of aromatic radicals.
- 4/B Higher-order condensed aromatics (e.g., terphenyls, quaterphenyls, triphenylene), representing advanced stages of aromatic condensation.
- 4/C Polycyclic aromatic structures (e.g., naphthalene, phenanthrene, fluorene derivatives), commonly formed during pyrolysis of aromatic polymers.
- 4/D Highly condensed aromatic structures, representing further recombination and condensation processes leading to complex multi-ring systems. This subgroup was excluded from subsequent quantitative evaluation because the detected compounds occurred only sporadically (≤2 samples) and therefore did not meet the prevalence threshold applied for the core dataset.

These subcategories describe structural relationships within the dataset and provide a framework for evaluating compositional patterns.

##### Occurrence and Quantitative Patterns A-AROM

A total of 15 A-AROM compounds were initially identified. Applying a prevalence threshold of detection in more than 6 out of 20 samples reduced the set to 9 frequently occurring compounds used for further evaluation. The A-AROM group exhibited considerable variability across samples in both compound counts and relative contributions. Total contributions ranged from approximately 0.18 to 2.60%, with the highest values observed in Vittel, Mattoni, Natura, and Poděbradka Proline, while no A-AROM compounds were detected in Römerquelle. Among the structural subcategories, polycyclic aromatic structures (4/C) represented the dominant fraction, followed by higher-order condensed aromatics (4/B) and biphenyl-type recombination products (4/A). Relative contributions varied across samples, with no consistent pattern observed for beverage type, bottle colour, or transformation-intensity categories.

To facilitate comparison, samples were grouped based on total A-AROM contributions and compound counts (Table S6). This grouping revealed differences in the extent of aromatic recombination patterns across bottles. Some samples, such as Römerquelle and Ondrášovka, showed minimal or no detectable A-AROM structures. In contrast, others, including Vittel, Mattoni, and Natura, exhibited higher counts and contributions, indicating more extensive formation of condensed aromatic structures under the applied analytical conditions. Variability was also observed within beverage categories. While clustering tendencies were present for samples with higher contributions, overall patterns remained heterogeneous and did not indicate a single dominant controlling factor.

**Figure 4:**
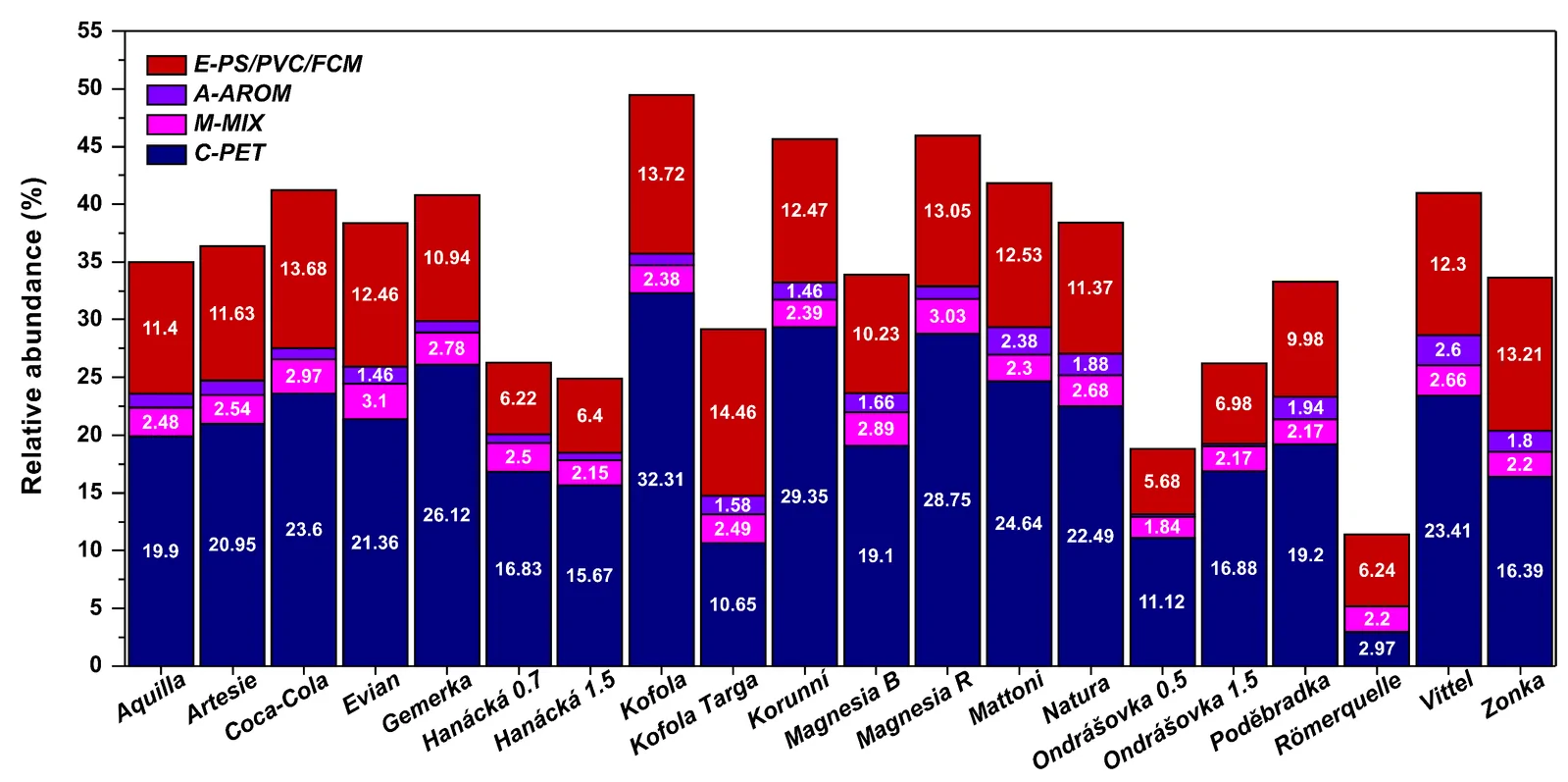
Relative contribution of marker groups across PET bottles expressed as percentage peak areas.

In summary, the A-AROM group reflects the formation of condensed aromatic structures during pyrolysis through the recombination and condensation of aromatic fragments. These compounds were detected in most analysed PET bottles, with substantial variability in both occurrence and relative contributions. Total concentrations ranged from 0.00 to 2.60%, with the highest values in Vittel, Mattoni, and Natura, while Römerquelle showed no detectable A-AROM compounds. Across all samples, classical PAH-like structures (4/C) represented the dominant fraction, followed by higher-order condensed aromatics (4/B), whereas biphenyl-type recombination products (4/A) contributed only marginally. The number of detected compounds per sample ranged from 0 to 10, indicating considerable variability in aromatic recombination patterns among bottles. No consistent trends were observed for beverage type, bottle colour, or nominal volume (Table S6). Figure 3C presents the distribution of condensed aromatic subgroup categories across PET bottles. Figure 4 summarises the overall distribution of the four principal diagnostic profile groups across PET bottles.

### 3.3. Relative Contribution and Multivariate Analysis of Marker Groups

To provide an integrated overview of the relative contributions of individual diagnostic profile groups across samples, the aggregated group data were visualised as stacked relative abundances (Figure 4). Across all PET bottles, PET transformation products (C-PET) accounted for the dominant fraction of the chemical profiles [45]. However, substantial variability was observed in the relative contributions of packaging-associated compounds (E-PS/PVC/FCM) and condensed aromatic structures (A-AROM), which together accounted for the main differences between samples [7,46].

Samples such as Mattoni, Vittel, and Kofola exhibited higher overall contributions of packaging-related inputs and aromatic structures, indicating increased chemical complexity. In contrast, bottles such as Römerquelle and Ondrášovka showed lower contributions of these groups and were dominated by C-PET [3,37]. The M-MIX group contributed only a minor and relatively stable fraction across all samples, consistent with its interpretation as a background component rather than a discriminative factor [39].

This compositional overview demonstrates that variability among commercial PET bottles is driven primarily by differences in packaging-related inputs and aromatic transformation behaviour. In contrast, PET transformation products constitute a relatively stable chemical background.

#### Multivariate Analysis and Composite Chemical Index (QI)

Principal component analysis (PCA) was performed using the four aggregated diagnostic profile groups (C-PET, E-PS/PVC/FCM, M-MIX, and A-AROM) to evaluate the dominant patterns of chemical variability among PET bottles. Before PCA, variables were autoscaled (z-score standardisation) to ensure comparable weighting of all diagnostic profile groups. The first two principal components explained 80.9% of the total variance (PC1 = 62.9%, PC2 = 18.0%). PC1 represented the dominant gradient of overall chemical complexity and packaging-related chemical burden, whereas PC2 reflected secondary differences in the relative balance between mixed-origin compounds (M-MIX) and aromatic transformation products (A-AROM).

**Figure 5:**
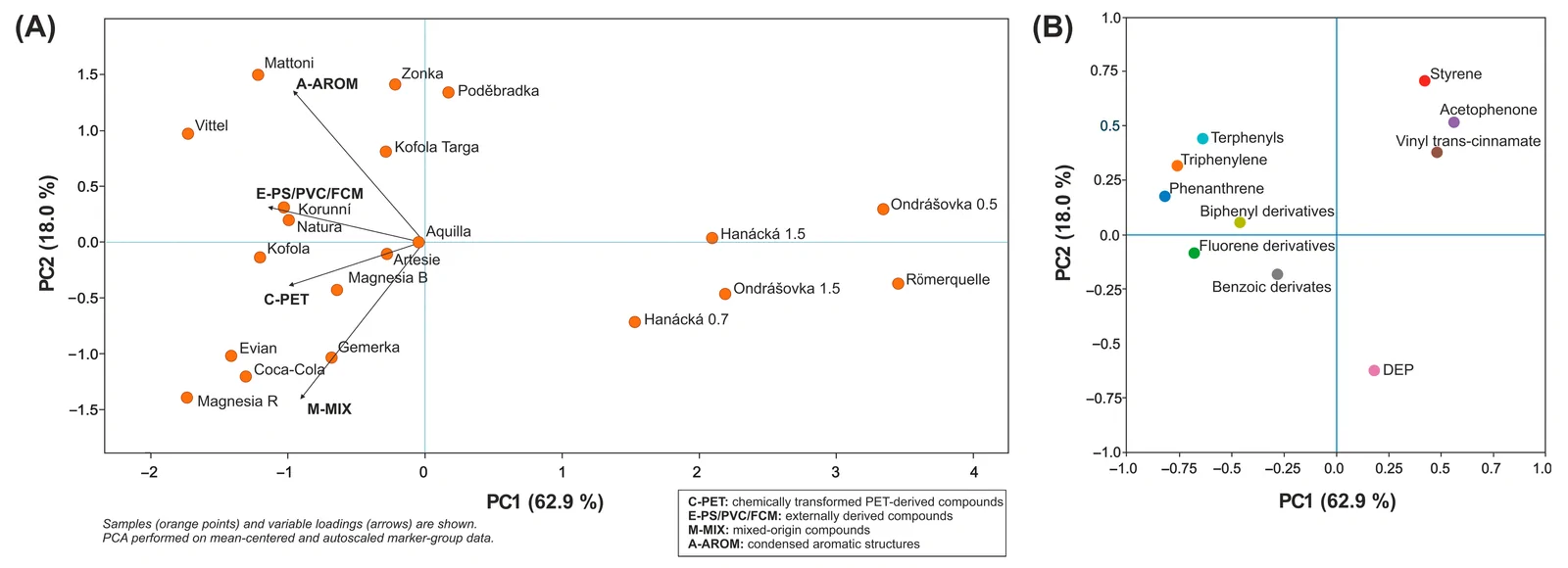
Principal component analysis (PCA) of PET bottle chemical profiles. (**A**) PCA score plot of 20 PET bottles based on four aggregated diagnostic profile groups (C-PET, E-PS/PVC/FCM, M-MIX, and A-AROM). PC1 explains 62.9%, and PC2 explains 18.0% of the total variance. Arrows indicate variable loadings; (**B**) PCA loading plot of selected compounds contributing to sample differentiation.

The PCA score plot (Figure 5A) showed a clear separation of bottles according to their overall chemical profiles. Samples characterised by lower contributions of packaging-associated compounds (E-PS/PVC/FCM) and aromatic transformation products, including Ondrášovka 0.5, Ondrášovka 1.5, Hanácka 1.5, Hanácka 0.7, and Römerquelle, formed a distinct group associated with chemically simpler profiles. In contrast, bottles such as Mattoni, Vittel, Korunní, Kofola, Coca-Cola, and Magnesia R showed higher contributions of E-PS/PVC/FCM and A-AROM, reflecting greater chemical complexity and a stronger influence of packaging-related inputs and transformation processes [3,46].

The distribution of diagnostic profile groups along the PCA axes indicated that E-PS/PVC/FCM, A-AROM, and C-PET contributed substantially to sample differentiation along PC1, whereas M-MIX contributed more strongly to PC2. The compound-level loading plot (Figure 5B) further highlighted the contribution of aromatic transformation products and packaging-associated compounds to sample separation. Compounds associated with aromatic condensation products, including phenanthrene and triphenylene, were associated primarily with PC1, whereas packaging-associated compounds such as styrene, acetophenone, and vinyl trans-cinnamate contributed more strongly to PC2. Diethyl phthalate (DEP) showed a distinct loading pattern consistent with its interpretation as a marker of packaging-related inputs. PC2 primarily reflected differences in the relative proportion of M-MIX versus A-AROM contributions. Samples with similar overall chemical complexity but different balances of aromatic and mixed-origin compounds were separated along this axis [20,32].

**Figure 6:**
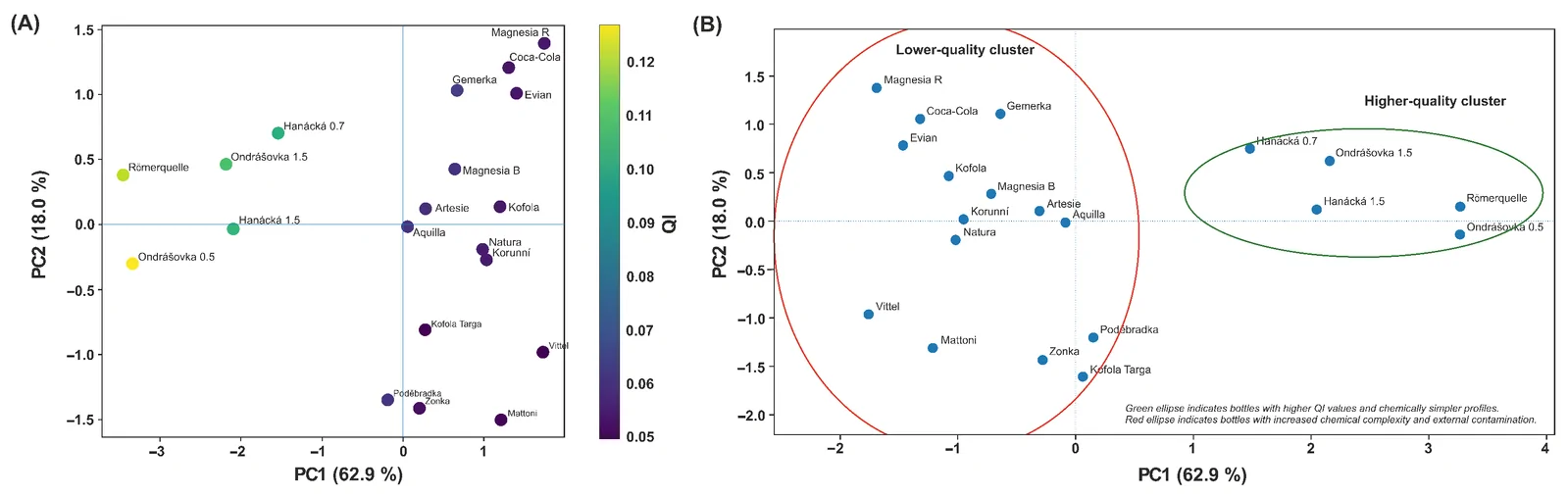
(**A**) PCA score plot of PET bottles coloured according to Quality Index (QI); (**B**) PCA score plot showing higher-quality (green ellipse) and lower-quality (red ellipse) bottle clusters based on QI.

To facilitate interpretation of overall chemical complexity, a composite Quality Index (QI) was used as an integrated descriptor of chemical profile quality (Figure 6). Higher QI values corresponded to bottles with lower contributions of packaging-associated compounds and aromatic transformation products, while lower QI values were associated with chemically more complex profiles. The distribution of QI values across the PCA space showed a strong relationship between QI and the primary PCA gradient. Bottles with high QI values clustered in the region associated with lower E-PS/PVC/FCM and A-AROM contributions, whereas low-QI bottles occupied the region associated with higher packaging-related chemical burden and aromatic transformation products. Although QI generally followed the same trend as PC1, it simplified multidimensional chemical variability into a single integrated descriptor and therefore did not fully reproduce the complete PCA structure.

Notably, bottles declared to contain recycled PET (rPET) were distributed across the full PCA space, indicating that nominal recycled content alone does not determine chemical profile characteristics. Instead, the observed variability appears to reflect differences in feedstock quality, recycling history, processing conditions, and packaging-related inputs [7,45]. In summary, PCA provided a robust framework for interpreting chemical variability among PET bottles. At the same time, QI served as a simplified complementary metric enabling direct comparison of overall chemical profile quality across samples. Both approaches consistently identified packaging-associated compounds and aromatic transformation products as major contributors to overall chemical complexity.

## 4. Discussion

### 4.1. PET Mass per Litre

The observed differences in PET mass per litre are consistent with previous life-cycle assessment (LCA) studies showing that bottle mass is determined primarily by structural design rather than polymer type or recycled content [24,25]. Higher material intensity in small-volume bottles (Figure 2A) reflects the disproportionate contribution of fixed structural components, such as the neck and base, which do not scale linearly with bottle volume.

These findings should also be interpreted in the context of ongoing lightweighting strategies aimed at reducing material consumption and environmental impacts associated with PET packaging [25,47]. However, no direct relationship was observed between PET mass per litre and the chemical patterns identified in this study (Figure 4, Figure 5 and Figure 6). Bottles with lower material intensity did not consistently exhibit lower chemical complexity or reduced contributions of the diagnostic profile groups associated with non-intentionally added substances (NIAS). This indicates that material efficiency and chemical profile characteristics represent largely independent aspects of PET bottle performance. In summary, PET mass per litre serves as a useful descriptor of material efficiency and design optimisation but should be interpreted independently of chemical profile characteristics when evaluating PET packaging quality.

### 4.2. Integrated Interpretation of Marker-Group Contributions

The relative contributions of diagnostic profile groups across PET bottles (Figure 4) provide an integrated overview of chemical composition patterns preceding detailed group-specific discussions. Across all samples, PET transformation products (C-PET) accounted for the dominant fraction of the chemical profiles, indicating that polymer degradation products are the primary component of pyrolysis-derived signals.

In contrast, packaging-associated compounds (E-PS/PVC/FCM) showed substantial variability between bottles and represented the principal source of inter-sample differences. This variability reflects differences in packaging-related inputs such as adhesives, coatings, and printing inks, highlighting the importance of packaging-related inputs in shaping NIAS profiles. Condensed aromatic structures (A-AROM), although present at lower absolute levels, also varied between samples and indicate differences in the propensity of materials to form higher-order aromatic structures under pyrolytic conditions. Mixed-origin compounds (M-MIX) contributed only a minor but stable fraction across all samples, supporting their interpretation as a background component of limited discriminative value.

The multivariate analysis further revealed a continuum of chemical complexity rather than discrete categories (Figure 5 and Figure 6). Bottles grouped within the higher-QI cluster were characterised by lower contributions of E-PS/PVC/FCM and A-AROM compounds, suggesting comparatively lower contributions of packaging-related inputs and lower transformation intensity. In contrast, bottles within the lower-QI cluster exhibited elevated levels of these components, indicating more complex chemical profiles associated with packaging-related inputs, additive inputs, and cumulative thermal history.

Importantly, this variability was not consistently associated with beverage type or declared recycled content. Instead, the observed differences appear to reflect variations in feedstock quality, processing conditions, and the heterogeneity of recycled PET input streams, consistent with previous studies identifying packaging-related inputs and recycled-stream variability as major drivers of chemical heterogeneity in recycled PET [7,11].

In this context, the Quality Index (QI) provides a simplified descriptor of chemical complexity, supporting comparative interpretation of multivariate patterns across heterogeneous PET materials. Although not intended as a quantitative measure of safety, QI enables the identification of samples with relatively higher or lower overall chemical complexity.

### 4.3. C-PET (Intrinsic PET Degradation)

The C-PET group represents PET transformation products and forms the dominant and most consistent component of the chemical profiles across all analysed bottles. The qualitative composition of the C-PET profile was highly stable, indicating that all samples share common transformation pathways irrespective of brand, bottle colour, or beverage type.

Terephthalic acid consistently represented the dominant transformation product, while biphenyls, aromatic aldehydes, terphenyls, and low-molecular-weight aromatics formed a characteristic suite of PET-derived transformation compounds. These structures arise from well-established degradation mechanisms, including ester bond scission and decarboxylation [32,37] followed by oxidation [45] and subsequent radical recombination during processing and recycling [3,20].

Despite this qualitative consistency, substantial quantitative differences were observed between bottles, with total C-PET contributions ranging from approximately 11 to 32% of total concentrations (Table S4). These differences reflect variation in transformation intensity rather than fundamental compositional changes. Biphenyls and terphenyls contributed most strongly to inter-sample variability, while terephthalic acid dominated the overall mass balance. Benzene and phenol were detected at low but relatively stable levels, consistent with their volatility and diffusion behaviour in PET matrices [36,48]. The observed quantitative variation defines a continuum of transformation intensity across samples, ranging from materials with limited thermal and oxidative exposure to those reflecting more extensive processing histories. Figure 3 illustrates this gradient, which aligns with the quantitative distribution reported in Table S4.

This gradient of transformation intensity suggests that quantitative differences in C-PET profiles primarily reflect cumulative thermal and oxidative exposure rather than intrinsic differences in polymer composition. Materials with lower contributions of higher-order aromatic compounds likely correspond to less processed PET, whereas higher contributions indicate increased transformation associated with repeated processing or more demanding manufacturing conditions. The apparent association with beverage categories, therefore, reflects differences in packaging design, material grade, and processing history rather than the influence of the contained product itself.

### 4.4. E-PS/PVC/FCM Group

The E-PS/PVC/FCM group represents the primary source of variability in the chemical profiles and reflects contributions from external materials rather than PET transformation processes. The substantial differences observed across samples confirm that packaging-related inputs, including labels, adhesives, coatings, and printing inks, play a decisive role in shaping NIAS profiles in PET bottles [7,49].

Among all subgroups, benzoate-type esters emerged as the most consistent and dominant contributors across the dataset. Their ubiquitous presence, irrespective of beverage type, bottle colour, or brand, supports the interpretation that they are persistent markers of packaging-related contamination rather than products of PET degradation [30,50]. These compounds are unlikely to form during pyrolysis, as their structures require pre-existing ester linkages, supporting their predominant association with adhesives, coatings, and ink systems [43]. Across the analysed samples, benzoate-type esters contributed the largest fraction of the E-PS/PVC/FCM group, with total concentrations ranging from approximately 6.2 to 14.5% (Table S5). Their persistence across all samples suggests limited removal efficiency during mechanical recycling and highlights a fundamental limitation in eliminating certain classes of NIAS [49].

Other subgroups (3/A and 3/B; see Section 3.2.3 and Table S5) contributed to differentiation between samples. Low-molecular-weight aromatics and styrenic compounds reflected inputs from styrenic materials and packaging components, while aromatic ketones were associated with photoinitiators and UV-curable coatings [41,42]. These compounds showed variability across bottles and contributed to differences related to packaging design, printing technologies, and processing history. This variability is reflected in Figure 4 and aligns with the quantitative patterns reported in Table S5. Among beverage categories, soft drinks exhibited the highest overall contributions (≈13.2–14.5%) and compound counts (19–22), reflecting the combined effects of aggressive matrices and more complex packaging systems. Flavoured mineral waters showed greater heterogeneity (≈5.7–13.1%; 14–21 compounds), whereas plain waters displayed lower variability but not necessarily lower contamination levels (≈6.2–12.5%; 8–21 compounds). These patterns further support the interpretation that packaging design and technological history may exert a stronger influence on chemical variability than the contained product itself [51].

Plasticiser-related compounds, although excluded from the core dataset due to low prevalence, represent a distinct and regulatorily significant subset within the E-PS/PVC/FCM group. The detection of compounds such as diethyl phthalate (DEP), DEHT, and DEHA indicates the presence of external ester-based additives within the analysed PET materials [44]. Plasticisers are of particular concern because they can migrate and, in some cases, have endocrine-disrupting properties [14,52]. Detected concentrations ranged from trace levels up to several per cent for individual compounds, with DEP showing the highest variability across samples. Their occurrence underscores the importance of considering not only dominant but also low-prevalence contaminants in assessing recycled PET, particularly with respect to potential migration under use conditions [53,54].

In summary, the E-PS/PVC/FCM group demonstrates that packaging-related inputs are a major determinant of chemical variability in PET bottles. While benzoate-type esters form a persistent baseline reflecting packaging-related inputs, variability in other subgroups and the presence of plasticisers reveal the combined influence of packaging complexity, processing history, and material heterogeneity. These findings emphasise that PET quality assessment cannot rely solely on PET transformation products but must also account for packaging-associated NIAS.

### 4.5. M-MIX

The M-MIX group comprises compounds of mixed or ambiguous literature associations that cannot be unequivocally attributed to PET transformation processes or packaging-related inputs. It reflects a transitional space between PET-derived transformation products and externally introduced substances, capturing background chemical complexity [7,49].

Across all samples, the M-MIX profile was highly consistent qualitatively and quantitatively, with total contributions ranging from approximately 1.8–3.1% (Tables S2 and S3), with most bottles clustering between 2.2 and 2.7%. Assigned compounds, including ethylene glycol mono- and dibenzoates, are not characteristic products of primary PET degradation and are predominantly associated with interactions between PET transformation intermediates and packaging-related inputs, such as adhesives, coatings, or inks. However, minor secondary formation during thermal processing cannot be excluded [7,38]. (E)-Stilbene was excluded from the core set due to its low prevalence, indicating that only a limited subset contributes consistently across samples.

In contrast to C-PET and E-PS/PVC/FCM, no systematic trends were observed with respect to beverage type, bottle colour, brand, or transformation-intensity categories. M-MIX compounds, therefore, appear to represent a stable background component of chemical complexity rather than primary drivers of inter-sample differentiation. Although M-MIX contributed only weakly to PCA separation, its inclusion supports a balanced interpretation of overall chemical profiles by preventing multivariate patterns from being dominated exclusively by transformation or packaging-related markers.

### 4.6. A-AROM (Condensed Aromatic Structures)

The A-AROM group provides insight into the formation of condensed aromatic structures during high-temperature pyrolysis of PET and associated materials. These compounds arise from radical recombination and secondary condensation of aromatic fragments generated during polymer decomposition and, therefore, reflect transformation behaviour under the applied analytical conditions rather than the direct presence of original contaminants [17]. Formation pathways of polycyclic and condensed aromatics during pyrolysis are well documented and may involve a wide range of aromatic precursors, including PET, styrenic materials, additives, and trace organic residues [18,22]. This interpretation is further supported by the recent micro-pyrolysis study of Perez and Toraman [55], which demonstrated that several aromatic compounds previously considered potential indicators of external inputs may also be generated directly from PET under controlled pyrolysis conditions. These findings highlight the limitations of assigning unique origins to individual pyrolysis products and reinforce the need for literature-informed, pattern-based interpretation of Py-GC/MS fingerprints [55].

Despite their analytical origin, the variability observed in A-AROM patterns across samples remains informative. Differences in total concentrations and compound counts (Table S6) indicate that PET bottles vary in their propensity to generate condensed aromatic structures under identical pyrolytic conditions. This behaviour likely reflects variations in polymer composition, additive content, prior thermal exposure, and incorporation of non-PET materials during recycling. Higher A-AROM contributions observed in selected samples, such as Vittel, Mattoni, and Natura, suggest a greater availability of aromatic precursors capable of undergoing recombination and condensation reactions [56]. No consistent relationship was observed with respect to beverage type or bottle colour, indicating that A-AROM formation is governed by multifactorial influences related to polymer history and input stream heterogeneity [11].

From a risk perspective, A-AROM compounds require cautious interpretation. Although some detected structures are chemically related to toxicologically relevant compound classes, their formation during pyrolysis does not imply their presence in the original material or their relevance for migration-based exposure. Pyrolysis–GC/MS is known to generate secondary transformation products that may not reflect the native chemical composition [57], and detected compounds may, therefore, represent analytical artefacts rather than substances relevant to consumer exposure [18,58].

Nevertheless, A-AROM compounds provide complementary analytical value by capturing aspects of chemical complexity related to the transformation potential of PET materials under thermal stress, which are not fully represented by PET transformation products (C-PET) or packaging-associated compounds (E-PS/PVC/FCM) [45]. When interpreted alongside these diagnostic profile groups, A-AROM patterns contribute to a more comprehensive characterisation of chemical variability in PET bottles and further support the rationale for the literature-informed, pattern-based interpretation framework proposed in the present study.

### 4.7. Integrated Interpretation

The distribution of diagnostic profile groups across PET bottles (Figure 4) confirms that PET transformation products (C-PET) form the dominant baseline of all profiles, whereas packaging-associated compounds (E-PS/PVC/FCM) and condensed aromatic structures (A-AROM) account for most inter-sample variability. In contrast, M-MIX compounds contribute a relatively stable background component with limited discriminative value.

PCA further revealed a continuum of chemical complexity rather than discrete categories (Figure 5 and Figure 6). Bottles with higher QI values were associated with lower contributions of packaging-associated compounds and aromatic transformation products. In contrast, lower-QI samples showed more complex profiles driven by packaging-related inputs, additive inputs, and thermal history. Importantly, this variability was not linked to declared recycled content but rather to differences in feedstock quality, processing conditions, and input-stream heterogeneity. These findings support the proposed literature-informed framework as a practical approach for comparative interpretation of Py-GC/MS fingerprints in recycled PET, complementing conventional compound-by-compound evaluation.

## 5. Conclusions

This study demonstrates that chemical variability in PET bottles appears to reflect the combined influence of PET transformation processes, packaging-related inputs, and transformation-related aromatic processes. While PET transformation products (C-PET) form a relatively consistent chemical baseline, variability across samples is primarily driven by packaging-associated compounds (E-PS/PVC/FCM) and aromatic transformation products.

The proposed literature-informed pattern-based interpretation framework, integrated with multivariate analysis, provides a systematic approach for interpreting complex chemical patterns in recycled PET. By distinguishing between PET transformation products, packaging-associated compounds, mixed-origin compounds, and condensed aromatic structures, this approach enables more robust evaluation of heterogeneous materials beyond conventional compound-by-compound analysis. Rather than assigning unique sources to individual compounds, the framework supports comparative interpretation of Py-GC/MS fingerprints based on diagnostic profile groups that represent predominant literature associations.

Importantly, the results show that chemical complexity is not determined solely by declared recycled PET content but is strongly influenced by feedstock quality, recycling history, packaging design, and processing conditions. These findings highlight the importance of controlling packaging-related contamination pathways and cumulative thermal exposure to improve the chemical quality of recycled PET materials. In summary, the proposed framework offers a practical and scalable tool for evaluating recycled PET in food-contact applications and supports the development of recycling strategies that address chemical quality beyond nominal rPET content. The framework is intended to evaluate relative chemical complexity and compositional variability rather than serve as a direct indicator of toxicological risk or regulatory compliance.

Future studies should focus on experimental validation of the proposed diagnostic profile groups using controlled reference materials and extending the framework to additional recycled polymer systems.

## Figures and Tables

**Table 1:** Characteristics of the analysed commercial PET beverage bottles, beverage categories, and publicly declared recycled PET (rPET) content.

ID	Bottle Name	Volume (L)	Bottle Colour	Flavour	Beverage Type	Declared rPET Content
PET1	Hanácká kyselka 1.5	1.50	Colourless	Orange	FMW	Estimated ≥25% rPET (EU requirement)
PET2	Hanácká kyselka 0.7	0.70	Colourless	Collagen addition	PW	
PET3	Ondrášovka 1.5	1.50	Green	Forest fruits	FMW	
PET4	Ondrášovka 0.5	0.50	Green	Lemon	FMW	
PET5	Mattoni	1.50	Green		PW	Manufacturer declares transition to 100% rPET for selected lines
PET6	Gemerka	1.50	Blue	Sea buckthorn	FMW	Estimated 25–30% rPET (EU requirement)
PET7	Poděbradka Prolinie	1.50	Colourless	Lemon	FMW	Manufacturer declaration: transition to 100% rPET
PET8	Artesie	1.50	Colourless	Orange	FMW	No public declaration
PET9	Aquila	1.50	Colourless		PW	Manufacturer declaration: transition to 100%rPET
PET10	Natura	1.50	Blue		PW	Manufacturer declaration: transition to 100%rPET
PET11	Magnesia Blue	1.50	Blue		PW	Manufacturer declaration: transition to 100%rPET
PET12	Vittel	0.70	Colourless		PW	Manufacturer declaration: transition to 100% rPET
PET13	Evian	0.50	Colourless		PW	Manufacturer declaration: 10% rPET(selected markets)
PET14	Korunní	1.50	Green		PW	Estimated 25–30% rPET (EU requirement)
PET15	Römerquelle	0.50	Green		PW	Manufacturer declaration: transition to 100% rPET (selected bottles)
PET16	Rajec	0.50	Colourless		PW	Estimated 25–30% rPET (EU requirement)
PET17	Kofola	0.50	Colourless	Sugar-free cola	SD	Manufacturer declaration: 25–30% rPET
PET18	Zonka	0.50	Colourless	Lemon	SD	No public declaration
PET19	Saguaro	0.50	Colourless	Forest fruits	SD	No public declaration
PET20	Coca-Cola	0.50	Colourless		SD	Manufacturer declaration: 100% rPET
PET21	Magnesia Red	1.50	Red	Raspberry	FMW	Manufacturer declaration: transition to 100% rPET
PET22	Kofola Targa	1.33	Red	Mandarin + passion fruit	SD	Estimated 25–30% rPET (EU requirement)

Abbreviations: FMW = flavoured mineral water; PW = plain/mineral water; SD = soft drink. Declared rPET content is based on publicly available manufacturer information and was used solely for sample categorisation. No independent verification of recycled PET content was performed.

## Data Availability

The original contributions presented in this study are included in the article/Supplementary Material. Further inquiries can be directed to the corresponding author.

## Supplementary Materials

The following supporting information can be downloaded at: https://www.mdpi.com/article/10.3390/polym18182194/s1. Table S1. Overview of PET bottle samples. Beverage type and rPET information. Table S2 Sources and characteristics of identified compounds in PET, including their removability and legislative risks. Table S3 Concentrations of identified compounds in PET; Table S4. C-PET compound groups and their contribution to chemical load; Table S5. Distribution of externally derived compounds (E-PS/PVC/FCM), including group composition, compound counts and relative abundances across PET bottle types; Table S6. Occurrence and relative contribution of condensed aromatic structures (A-AROM) in PET bottles.

## Abbreviations

The following abbreviations are used in this manuscript:

A-AROM	Condensed aromatic structures
BTX	Benzene, toluene, and xylene
C-PET	Chemically transformed PET-derived compounds
DEHA	Di(2-ethylhexyl) adipate
DEHT	Di(2-ethylhexyl) terephthalate
DEP	Diethyl phthalate
DOI	Digital object identifier
E-PS/PVC/FCM	Externally derived compounds associated with polystyrene, polyvinyl chloride, and food-contact materials
ESF	European Social Fund
FCM	Food-contact material
GC	Gas chromatography
GC/MS	Gas chromatography–mass spectrometry
LCA	Life-cycle assessment
M-MIX	Mixed-origin compounds
MSD	Mass-selective detector
NIAS	Non-intentionally added substances
PAH	Polycyclic aromatic hydrocarbon
PC1	First principal component
PC2	Second principal component
PCA	Principal component analysis
PET	Polyethylene terephthalate
PS	Polystyrene
PVC	Polyvinyl chloride
Py-GC/MS	Pyrolysis–gas chromatography–mass spectrometry
QI	Quality Index
rPET	Recycled polyethylene terephthalate
