# Supplementary material for: Chemical Variability of Recycled PET Bottles Driven by Packaging Inputs and Processing History: A Marker-Based Multivariate Approach
Source: Polymers (Basel). 2026 Sep 8;18(18):2194. doi: 10.3390/polym18182194 (PMC13611611; doi:10.3390/polym18182194)
Supplement: Supplementary file 1 [file polymers-18-02194-s001.zip › polymers-4460553-supplementary.pdf]

SUPPLEMENTARY MATERIAL

Table S1. Overview of PET bottle samples. beverage type. and rPET information.

ID	Bottle name	Volume (L)	Weight of empty PET bottle (g)	Bottle colour	Flavour	Beverage type	Manufacturer/Producer	Brand owner/Group	rPET information
PET 1	Hanácká kyselka 1.5	1.50	30.141	Colourless	Orange	FMW	Mattoni 1873 a.s. (Karlovy Vary, Czech Republic)	Mattoni 1873 a.s. (Karlovy Vary, Czech Republic)	In accordance with EU legislation (Directive (EU) 2019/904). PET beverage bottles placed on the EU market must contain at least 25% recycled PET by 2025
PET 2	Hanácká kyselka 0.7	0.70	26.431	Colourless	Collagen addition	PW	Mattoni 1873 a.s. (Karlovy Vary, Czech Republic)	Mattoni 1873 a.s. (Karlovy Vary, Czech Republic)	
PET 3	Ondrášovka 1.5	1.50	23.676	Green	Forest fruits	FMW	Kofola a.s. (Krnov, Czech Republic)	Kofola ČeskoSlovensko a.s. (Ostrava, Czech Republic)	
PET 4	Ondrášovka 0.5	0.50	12.122	Green	Lemon	FMW	Kofola a.s. (Krnov, Czech Republic)	Kofola ČeskoSlovensko a.s. (Ostrava, Czech Republic)	
PET 5	Mattoni	1.50	32.881	Green		PW	Mattoni 1873 a.s. (Karlovy Vary, Czech Republic)	Mattoni 1873 a.s. (Karlovy Vary, Czech Republic)	Manufacture declares transition to 100% rPET for selected lines
PET 6	Gemerka	1.50	28.141	Blue	Sea buckthorn	FMW	BUDIŠ a.s. (Tornaľa, Slovakia)	BUDIŠ a.s. (Dubové, Slovakia)	25-30% in accordance with EU legislation (Directive EU 2019/904)
PET 7	Poděbradka Prolinie	1.50	28.514	Colourless	Lemon	FMW	Mattoni 1873 a.s. (Karlovy Vary, Czech Republic)	Mattoni 1873 a.s. (Karlovy Vary, Czech Republic)	Manufacture declares transition to 100% rPET for selected lines
PET 8	Artesie	1.50	23.736	Colourless	Orange	FMW	VESETA spol. s r.o. (Braškov, Czech Republic)	VESETA spol. s r.o. (Braškov, Czech Republic)	No information
PET 9	Aquila	1.50	28.440	Colourless		PW	Mattoni 1873 a.s. (Karlovy Vary, Czech Republic)	Mattoni 1873 a.s. (Karlovy Vary, Czech Republic)	Manufacture declares transition to 100%r-PETfor selected lines
PET 10	Natura	1.50	30.376	Blue		PW	Coca-Cola HBC Česko a Slovensko, s.r.o. (Prague, Czech Republic)	The Coca-Cola Company (Atlanta, GA, USA)	100% rPET
PET 11	Magnesia Blue	1.50	30.448	Blue		PW	Mattoni 1873 a.s. (Karlovy Vary, Czech Republic)	Mattoni 1873 a.s. (Karlovy Vary, Czech Republic)	Selected Magnesia product declared 100% rPET
PET 12	Vittel	0.70	18.099	Colourless		PW	Nestlé Waters Supply Est (Issy-les-Moulineaux, France)	Nestlé S.A. (Vevey, Switzerland)	100% rPET
PET 13	Evian	0.50	12.522	Colourless		PW	Société Anonyme des Eaux Minérales d'Évian (Évian-les-Bains, France)	Danone S.A. (Paris, France)	Danone declares transition to 10%r-Pet for selected markets
PET 14	Korunní	1.50	26.674	Green		PW	Kofola a.s. (Krnov, Czech Republic)	Kofola ČeskoSlovensko a.s. (Ostrava, Czech Republic)	25-30% in accordance with EU legislation (Directive EU 2019/904)
PET 15	Römerquelle	0.50	16.054	Green		PW	Coca-Cola HBC Austria GmbH (Vienna, Austria)	The Coca-Cola Company (Atlanta, GA, USA)	Coca-Cola declared 100% rPET for selected small bottles
PET 16	Rajec	0.50	19.859	Colourless		PW	Kofola a.s. (Rajecká Lesná, Slovakia)	Kofola ČeskoSlovensko a.s. (Ostrava, Czech Republic)	25-30% in accordance with EU legislation (Directive EU 2019/904)
PET 17	Kofola	0.50	43.200	Colourless	Sugar-free cola	SD	Kofola a.s. (Krnov, Czech Republic)	Kofola ČeskoSlovensko a.s. (Ostrava, Czech Republic)	Kofola declared a 25-30% in part of its portfolio
PET 18	Zonka	0.50	19.627	Colourless	Lemon	SD	"ZON" spol. s r.o. (Třebíč, Czech Republic)	"ZON" spol. s r.o. (Třebíč, Czech Republic)	No public declaration of rPET use for this product
PET 19	Saguaro	0.50	12.106	Colourless	Forest fruits	SD	Lidl Stiftung & Co. KG (Neckarsulm, Germany) / Schwarz Group (Neckarsulm, Germany)	Lidl Stiftung & Co. KG (Neckarsulm, Germany) / Schwarz Group (Neckarsulm, Germany)	No public declaration of rPET use for Saguaro bottles
PET 20	Coca-Cola	0.50	20.773	Colourless		SD	Coca-Cola HBC Česko a Slovensko, s.r.o. (Prague, Czech Republic)	The Coca-Cola Company (Atlanta, GA, USA)	100% rPET publicity declared for 0.5L bottles in CZ/SK
PET 21	Magnesia Red	1.50	31.500	Red	Raspberry	FMW	Mattoni 1873 a.s. (Karlovy Vary, Czech Republic)	Mattoni 1873 a.s. (Karlovy Vary, Czech Republic)	Manufacture declares transition to 100% rPET for selected lines
PET 22	Kofola Targa	1.33	35.664	Red	Mandarin + passion fruit	SD	Kofola a.s. (Krnov, Czech Republic)	Kofola ČeskoSlovensko a.s. (Ostrava, Czech Republic)	25-30% in accordance with EU legislation (Directive EU 2019/904)

Explanation: FMW = Flavoured mineral water; PW = Plain/mineral water; SD = Soft drink/soda.

Table S2. Sources, and characteristics of identified compounds in PET, including their removability and legislative risks.

Group of chemical compounds	Group	Compound	Origin group	Typical source	Removability	Regulatory risk (Beverage PET)	Marker
1/A Biphenyls and biphenyl-based derivatives	1/A	1,1′-Biphenyl	C-PET	Thermal stress;MOAH/PS-related aromatics	Moderate	Moderate–high	Aromatic condensation marker
	1/A	(1,1′-Biphenyl)-2,2′-dicarboxaldehyde	C-PET	In-situ oxidation/Condensation of PET aromatics	Low	High	PET thermal degradation marker
	1/A	2-Methyl-1,1′-biphenyl	C-PET	Aromatic condensation under thermal stress	Low	Moderate	PET thermal degradation marker
	1/A	4-Ethenyl-1,1′-biphenyl	C-PET	Condensed aromatic with vinyl substitution	Low	Moderate	Aromatic condensation marker
	1/A	4-Formyl-1,1′-biphenyl	C-PET	In-situ oxidation of PET aromatics	Low	High	PET thermal degradation marker
	1/A	4-Methyl-1,1′-biphenyl	C-PET	PET-derived aromatic transformed under heat	Low	Moderate	PET thermal degradation marker
1/B Aromatic aldehydes	1/B	Benzaldehyde	C-PET	Oxidation of PET aromatics/Ink components	Moderate	Moderate	PET oxidation marker
	1/B	Phenylacetaldehyde	C-PET	Oxidation of styrene/Ethylbenzene (Inks/Adhesives);Styrenic contamination associated with rPET	Moderate	Moderate–high	PET-relevant styrenic contamination marker
1/C Diphenylmethane	1/C	Diphenylmethane	C-PET	Secondary aromatic radical recombination	Low	Moderate	Secondary aromatic recombination marker
1/D Terphenyls	1/D	m-Terphenyl	C-PET	Advanced aromatic condensation	Very low	Moderate–high	Advanced aromatic condensation marker
1/E Benzoic acid and oxygenated derivatives	1/E	2-(1-Oxopropyl)benzoic acid	C-PET	Oxidised PET aromatics	Low	Moderate–high	PET oxidation marker
	1/E	4-Ethylbenzoic acid	C-PET	Oxidation of alkylated aromatics	Moderate	Moderate	Alkylated benzoic acid marker
	1/E	4-Methylbenzoic acid	C-PET	Oxidation of alkylated aromatics	Moderate	Moderate	Alkylated benzoic acid marker
	1/E	Benzoic acid	C-PET	Oxidation of PET aromatics	Moderate	Moderate	Primary PET degradation marker
	1/E	Methyl α-oxobenzoate	C-PET	Oxidised aromatics/Ink	Low	Moderate–high	Esterified oxidation marker
	1/E	Ethyl α-oxobenzoate	C-PET	Oxidised aromatics/Ink	Low	Moderate–high	Esterified oxidation marker
1/F Terephthalic acid (free monomer)	1/F	Terephthalic acid	P-PET	PET hydrolysis/Backbone cleavage	n.a.	Low–moderate	PET hydrolysis marker
1/G Secondary aromatic ketones	1/G	2-Methyl-1-(4-methylphenyl)propan-1-one	C-PET	Secondary aromatic ketone from rPET	Moderate	Low–moderate	rPET-related aromatic ketone
1/H C-PET/E (mixed origin: phenol and benzene)	1/H	Benzene	P-PET/E	PS/PVC pyrolysis;Occasional process-related	Moderate	High	Ubiquitous volatile aromatic (Non-diagnostic)
	1/H	Phenol	P-PET/E	Phenolic antioxidants;Thermal/Oxidative degradation;Ink	Moderate	Moderate–high	Phenolic antioxidant degradation marker
2/A Aromatic benzoate esters	2/A	Di(benzoyloxy)ethane	M-MIX	Benzoate-based ink/Adhesive components;Secondary reactions	Low	Moderate–high	Plasticizer/Adhesive-related contaminant
	2/A	2-Hydroxyethyl benzoate	M-MIX	Benzoate-based ink/Adhesive components;Secondary reactions	Moderate	Moderate	Plasticizer/Adhesive-related contaminant
2/B Stilbene-Type Aromatic Hydrocarbon	2/B	trans-Stilbene	M-MIX	PS contamination;Degradation of styrenic aromatics	Moderate	Moderate–high	Mixed-origin aromatic marker (Styrenic/Additive-related
3/A Low-molecular-weight aromatic hydrocarbons	3/A	1-Propynylbenzene	E-PS/PVC/FCM	PS pyrolysis/Styrenic degradation	Low	High	Styrenic/Solvent-related contamination
	3/A	1,3-Divinylbenzene	E-PS/PVC/FCM	PS/Styrenic chemistry	Moderate	High	Styrenic external contaminant
	3/A	1,4-Divinylbenzene	E-PS/PVC/FCM	PS/Styrenic chemistry	Moderate	High	Styrenic external contaminant
	3/A	2-Methylstyrene	E-PS/PVC/FCM	Alkylstyrene (PS)	Moderate	High	Styrenic external contaminant
	3/A	3-Methylstyrene	E-PS/PVC/FCM	Alkylstyrene (PS)	Moderate	High	Styrenic external contaminant
	3/A	4-Ethylstyrene	E-PS/PVC/FCM	Alkylstyrene (PS)	Moderate	High	Styrenic external contaminant
	3/A	4-Methylstyrene	E-PS/PVC/FCM	Alkylstyrene (PS)	Moderate	High	Styrenic external contaminant
	3/A	Cyclopropylbenzene	E-PS/PVC/FCM	PS pyrolysis/Styrenic degradation	Low	High	Styrenic pyrolysis marker
	3/A	Ethylbenzene	E-PS/PVC/FCM	PS/Styrenic contamination	High	Low–moderate	Styrenic contamination marker
	3/A	Indane	E-PS/PVC/FCM	MOAH/Pyrolysis	Moderate	Moderate–high	MOAH-related aromatic marker
	3/A	para-Xylene	E-PS/PVC/FCM	MOAH/Solvents	Moderate	Moderate	Solvent-related aromatic marker
	3/A	Styrene	E-PS/PVC/FCM	PS contamination	Moderate	High	Primary styrenic contamination marker
	3/A	Toluene	E-PS/PVC/FCM	Solvents/MOAH/Inks	Moderate	Moderate	Solvent-related contamination
3/B Aromatic ketones and related carbonyl compounds	3/B	1-(1,1′-Biphenyl-4-yl)ethan-1-one	C-PET	Functionalised condensed aromatic	Low	Moderate	UV-curable coating-related aromatic
	3/B	1-(4-Ethylphenyl)ethan-1-one	E-PS/PVC/FCM	Inks/Coatings	Moderate	High	Photoinitiator/Coating marker
	3/B	2-(Methoxymethyl)-2-phenyl-1,3-dioxolane	E-PS/PVC/FCM	Solvents/Inks/Adhesives	Low	High	Printing ink/Solvent marker
	3/B	2,2′,4′,6′-Tetramethylpropiophenone	E-PS/PVC/FCM	Inks/Coatings	Moderate	High	Specialty photoinitiator marker
	3/B	4-(N-Methyl-N-methoxy)indane-1-carboxamide	E-PS/PVC/FCM	Misuse (pharma/Agro/Technical)	Low	High	Indane-based external contaminant
	3/B	Acetophenone	E-PS/PVC/FCM / P-PET	Inks/Photoinitiators+process-related	Moderate	Moderate–high	Photoinitiator/Printing ink marker
	3/B	Fluoren-9-one	E-PS/PVC/FCM	Oxidation of fluorene	Low	Critical	Oxidation product of external aromatics
3/C Benzoate-type esters	3/C	4-Vinylbenzoic acid	E-PS/PVC/FCM	Coatings/Ink monomers	Low	High	Oxidised vinylaromatic marker
	3/C	trans-Vinyl cinnamate	E-PS/PVC/FCM	Inks,Coatings,UV-curable;PS-related external contaminant	Low	High	Vinylaromatic/Cinnamate marker
	3/C	Vinyl benzoate	E-PS/PVC/FCM	Lacquers/Adhesives/Inks	Low	High	Vinylaromatic ester marker
	3/C	2,3-Dichlorophenyl 4-butylbenzoate	E-PS/PVC/FCM	Specialty ink/Coating systems	Low	High	Chlorinated aromatic ester
	3/C	2,5-Chlorophenyl 4-butylbenzoate	E-PS/PVC/FCM	Specialty ink/Coating systems	Low	High	Chlorinated aromatic ester
	3/C	4-Cyanophenyl 4-ethylbenzoate	E-PS/PVC/FCM	Specialty ink/Coating systems	Low	High	Cyanophenyl ester marker
	3/C	4-Cyanophenyl meta-toluate	E-PS/PVC/FCM	Specialty ink/Coating systems	Low	High	Cyanophenyl ester marker
	3/C	4-Cyanophenyl 4-propylbenzoate	E-PS/PVC/FCM	Specialty ink/Coating systems	Low	High	Cyanophenyl ester marker
3/D Plasticisers and external aromatic esters	3/D	Bis(2-ethylhexyl) terephthalate (DEHT)	E-PS/PVC/FCM	PVC/Inks additive;Not PET-derived	Low	High	Terephthalate plasticiser marker
	3/D	Bis(2-methylpropyl) phthalate	E-PS/PVC/FCM	Phthalate plasticiser	Low	High	Phthalate marker
	3/D	Bis(2-ethylhexyl) adipate (DEHA)	E-PS/PVC/FCM	PVC/Ink plasticiser;External origin	Low	High	Adipate marker
	3/D	Diethyl phthalate (DEP)	E-PS/PVC/FCM	Plasticiser/PVC/Adhesives/Inks	Low	High	Phthalate marker
	3/D	Cyclobutyl ethyl phthalate	E-PS/PVC/FCM	Specialty plasticiser	Low	High	Non-PET aromatic ester marker
	3/D	Diethylene glycol dibenzoate	E-PS/PVC/FCM	FCM/PVC/PU/Inks additives	Moderate	Moderate	Aromatic polyester-type plasticiser marker
	3/D	Phenyl 4-butylbenzoate	E-PS/PVC/FCM	Inks/Coatings/Adhesives	Low	Moderate–high	Aromatic ester from inks/Coatings
3/F Other additive-related markers	3/E	Triphenylphosphine oxide	E-PS/PVC/FCM	Catalysts,Stabilisers,Ink/Coating systems	Low	Moderate	Technical-aid contamination marker
4/A Biphenyl-type recombination products	4/A	1,2′-Binaphthalene	C-PAH/MOAH	Aromatic recombination of PAH fragments	Very low	High	Advanced aromatic condensation;Severe thermal stress
	4/A	2,2′-Binaphthalene	C-PAH/MOAH	Radical recombination of PAH fragments	Low	Critical	Highly condensed aromatics;Extreme thermal stress
	4/A	2,2′-Dimethyl-1,1′-biphenyl	C-PAH/MOAH	Aromatic radical recombination;High-temperature condensation;MOAH contamination	Low	High	Advanced aromatic condensation;Poor input quality;Excessive thermal stress
4/B Higher-order condensed aromatics	4/B	3′-Methyl-1,1′:4′,1″-terphenyl	C-PAH/MOAH	Secondary aryl–aryl condensation;Pyrolytic aromatization	Low	Critical	Highly condensed aromatics;Severe thermal degradation
	4/B	meta,meta-Quaterphenyl	C-PAH/MOAH	High-order aryl–aryl condensation	Very low	High	Extreme degradation;Long residence time;MOAH contamination
	4/B	ortho-Terphenyl	C-PAH/MOAH	Pyrolytic aromatization of PET/PS;MOAH contamination	Low	Critical	Advanced condensation;Low sorting quality
	4/B	Triphenylene	C-PAH/MOAH	PAH-type condensation;MOAH-rich materials	Low	Critical	Strong MOAH marker;High-temperature aromatization
4/C Classical PAHs and PAH-like structures	4/C	8,9-Dihydro-7H-cyclopenta[a]pyrene	C-PAH/MOAH	PAH;Pyrolysis of mixed polymers	Low	Critical	High-toxicity PAH marker;Extreme temperature/MOAH
	4/C	9-Methylene-9H-fluorene	C-PAH/MOAH	PAH-like condensation;MOAH-rich materials	Low	Critical	PAH-like signature;Mixed polymer input
	4/C	(E,E)-1,4-Diphenyl-1,3-butadiene	C-PAH/MOAH	PAH precursor;Styrenic/Mixed polymer inputs;MOAH-rich contamination	Low	High	PAH-like condensate;Mixed polymer input;High-temperature processing
	4/C	Naphthalene	C-PAH/MOAH	PAH;Pyrolysis product of PET/PS;MOAH	Moderate	Critical	External MOAH contamination;Pyrolytic signature
	4/C	1-Phenylnaphthalene	C-PAH/MOAH	PAH-like condensation;MOAH-rich oils/Inks	Low	Critical	Advanced aromatic condensation;MOAH contamination
	4/C	Phenanthrene	C-PAH/MOAH	PAH;Pyrolysis of mixed plastics	Low	Critical	Strong PAH marker;Unsuitable input material
4/D Highly condensed MOAH like aromatics	4/D	2-Methyl-3H-benz[e]indene	C-PAH/MOAH	MOAH-like aromatic fractions;Pyrolytic aromatization	Low	Critical	MOAH contamination;Condensed aromatics
	4/D	Benzene-tetrakis(cyclobutanyl)	C-PAH/MOAH	Highly condensed MOAH-like aromatic structures	Low	Critical	Strong MOAH marker;Contamination from oils/Inks

Table S3. Concentrations of identified compounds in PET

Group	Compound	Hanácká kyselka 1.5	Hanácká kyselka 0.7	Mattoni	Poděbradka ProLinie	Aquilla	Magnesia Blue	Magnesia Red	Ondrášovka 1.5	Ondrášovka 0.5	Korunní	Kofola	Zonka	Kofola Targa	Gemerka	Artesie	Natura	Vittel	Evian	Römerquelle	Coca-Cola
1/A	1.1′-Biphenyl	n.a.	n.a.	2.79	2.50	2.58	2.85	3.39	n.a.	1.58	2.81	3.41	3.94	3.35	2.78	2.72	2.81	2.97	2.47	n.a.	2.09
1/A	(1.1′-Biphenyl)-2.2′-dicarboxaldehyde	0.16	0.14	n.a.	n.a.	n.a.	1.63	0.17	0.09	n.a.	3.21	3.87	0.13	0.24	2.33	n.a.	2.00	2.41	2.06	0.51	0.17
1/A	2-Methyl-1.1′-biphenyl	0.016	n.a.	n.a.	n.a.	n.a.	n.a.	n.a.	n.a.	n.a.	n.a.	0.19	0.50	0.28	n.a.	n.a.	n.a.	n.a.	0.14	n.a.	0.18
1/A	4-Ethenyl-1.1′-biphenyl	0.21	n.a.	0.27	0.62	0.22	0.19	0.33	n.a.	0.20	0.17	0.15	0.18	0.13	0.22	0.21	0.23	0.27	0.18	n.a.	0.13
1/A	4-Formyl-1.1′-biphenyl	n.a.	0.22	n.a.	0.07	n.a.	n.a.	0.08	0.19	n.a.	0.08	0.09	0.08	0.06	n.a.	0.04	0.08	n.a.	n.a.	n.a.	0.07
1/A	4-Methyl-1.1′-biphenyl	0.09	0.08	0.10	n.a.	0.19	0.27	0.50	n.a.	n.a.	0.32	0.25	0.36	0.37	n.a.	n.a.	0.19	0.28	0.21	n.a.	0.32
1/B	Benzaldehyde	0.19	0.21	0.09	0.08	0.09	0.10	0.18	0.24	0.06	0.15	0.15	0.18	0.17	0.10	0.10	0.10	0.11	0.12	0.08	0.15
1/B	Phenylacetaldehyde	n.a.	n.a.	0.27	0.21	0.23	0.23	0.37	n.a.	0.11	0.29	0.35	0.43	0.38	0.23	0.22	0.26	0.27	0.27	n.a.	0.36
1/C	Diphenylmethane	n.a.	n.a.	0.20	0.16	0.15	0.14	n.a.	n.a.	n.a.	0.20	n.a.	n.a.	n.a.	0.17	0.13	0.16	0.17	n.a.	n.a.	n.a.
1/D	m-Terphenyl	0.19	0.21	0.61	0.52	0.47	0.50	0.38	0.323	0.21	0.12	0.73	0.32	0.15	0.52	0.44	0.50	0.51	0.43	n.a.	0.24
1/E	2-(1-Oxopropyl)benzoic acid	0.75	0.65	0.36	0.18	0.25	0.11	1.19	0.47	0.26	0.24	1.92	4.46	0.36	4.61	0.36	0.16	0.29	0.35	0.16	0.25
1/E	4-Ethylbenzoic acid	0.26	0.31	0.36	0.18	0.25	0.23	0.63	0.28	0.25	0.61	0.69	1.02	0.73	0.35	0.15	0.24	0.15	0.32	0.26	0.51
1/E	4-Methylbenzoic acid	0.29	0.32	1.09	0.68	0.90	0.65	1.36	0.17	0.30	1.14	1.32	1.83	1.48	0.77	1.02	0.91	0.98	0.87	0.52	1.14
1/E	Benzoic acid	12.56	13.65	16.98	12.27	13.35	11.03	18.61	14.05	6.97	18.39	17.63	1.82	2.32	13.13	14.44	13.22	13.67	12.75	0.74	16.53
1/E	Methyl α-oxobenzoate	n.a.	n.a.	n.a.	n.a.	n.a.	n.a.	0.18	n.a.	n.a.	n.a.	n.a.	0.14	0.19	n.a.	0.10	n.a.	n.a.	n.a.	n.a.	n.a.
1/E	Ethyl α-oxobenzoate	n.a.	n.a.	n.a.	n.a.	0.11	n.a.	n.a.	0.12	n.a.	n.a.	0.21	n.a.	n.a.	n.a.	n.a.	0.12	0.12	0.15	n.a.	0.18
1/F	Terephthalic acid	0.63	0.85	0.96	1.32	0.63	0.67	0.41	0.62	0.40	0.86	0.69	0.49	0.60	0.54	0.50	0.82	0.76	0.70	0.52	0.74
1/G	2-Methyl-1-(4-methylphenyl)propan-1-one	0.16	n.a.	0.59	0.47	0.50	0.46	0.72	n.a.	0.25	0.58	0.75	0.78	0.79	n.a.	0.49	0.56	0.60	0.56	n.a.	0.76
1/H	Benzene	0.16	0.17	0.12	0.06	0.20	0.18	0.36	0.16	0.19	0.18	0.25	0.33	0.28	0.05	0.16	0.36	0.14	0.36	0.17	0.11
1/H	Phenol	0.02	0.02	0.03	0.04	0.03	n.a.	0.05	0.03	n.a.	0.03	0.04	0.04	0.05	n.a.	0.02	n.a.	0.04	0.03	n.a.	0.04
2/A	Di(benzoyloxy)ethane	1.36	1.65	1.89	1.70	2.06	2.40	2.13	1.25	1.12	1.76	1.76	1.33	1.80	2.19	2.01	2.24	2.08	2.49	0.60	2.27
2/A	2-Hydroxyethyl benzoate	0.79	0.85	0.41	0.47	0.41	0.49	0.90	0.92	0.72	0.63	0.62	0.86	0.69	0.59	0.53	0.44	0.57	0.60	1.59	0.70
2/B	trans-Stilbene	n.a.	n.a.	n.a.	n.a.	0.12	n.a.	0.16	n.a.	n.a.	0.13	0.11	0.15	0.13	n.a.	0.12	n.a.	n.a.	0.09	n.a.	n.a.
3/A	1-Propynylbenzene	n.a.	n.a.	0.04	n.a.	0.03	n.a.	0.05	0.05	n.a.	0.04	0.05	0.16	0.05	n.a.	0.03	n.a.	0.04	0.04	n.a.	0.06
3/A	1.3-Divinylbenzene	n.a.	n.a.	n.a.	0.03	0.03	n.a.	0.07	n.a.	n.a.	n.a.	n.a.	0.07	0.05	n.a.	n.a.	0.03	0.04	0.03	n.a.	n.a.
3/A	1.4-Divinylbenzene	n.a.	n.a.	0.03	n.a.	n.a.	n.a.	n.a.	0.02	n.a.	0.04	0.05	n.a.	n.a.	n.a.	0.03	n.a.	n.a.	n.a.	n.a.	0.04
3/A	2-Methylstyrene	n.a.	n.a.	0.03	n.a.	n.a.	n.a.	n.a.	n.a.	n.a.	n.a.	n.a.	0.13	0.05	n.a.	n.a.	0.04	0.04	n.a.	n.a.	n.a.
3/A	3-Methylstyrene	n.a.	n.a.	n.a.	0.04	n.a.	n.a.	n.a.	n.a.	n.a.	n.a.	n.a.	n.a.	n.a.	0.04	0.03	n.a.	n.a.	n.a.	n.a.	0.04
3/A	4-Ethylstyrene	0.02	n.a.	0.03	n.a.	0.03	n.a.	0.05	0.015	n.a.	0.04	0.05	0.05	n.a.	n.a.	n.a.	n.a.	n.a.	0.04	n.a.	n.a.
3/A	4-Methylstyrene	n.a.	n.a.	0.04	0.03	n.a.	n.a.	0.04	n.a.	n.a.	n.a.	0.04	0.04	0.05	n.a.	0.03	0.03	n.a.	n.a.	n.a.	0.05
3/A	Cyclopropylbenzene	n.a.	0.03	0.07	n.a.	0.06	0.06	0.09	0.06	0.03	0.07	0.10	0.05	0.10	0.06	0.06	n.a.	0.07	0.07	n.a.	0.09
3/A	Ethylbenzene	0.06	n.a.	0.10	0.10	0.10	0.10	0.16	0.025	0.05	0.13	0.14	0.16	0.16	0.09	0.14	0.11	0.11	0.10	n.a.	0.13
3/A	Indane	0.03	0.03	0.02	0.02	0.02	0.06	0.02	0.02	0.01	0.02	0.03	0.04	0.01	0.02	0.02	0.07	0.03	0.03	0.02	0.06
3/A	para-Xylene	n.a.	0.05	n.a.	0.03	n.a.	n.a.	n.a.	n.a.	n.a.	n.a.	0.02	0.03	n.a.	n.a.	n.a.	n.a.	n.a.	n.a.	n.a.	n.a.
3/A	Styrene	0.16	0.25	0.41	0.34	0.37	0.37	0.61	0.21	0.20	0.50	0.54	0.56	0.60	0.38	0.41	0.42	0.46	0.46	n.a.	0.60
3/A	Toluene	0.32	0.28	0.41	0.35	0.36	0.40	0.39	0.62	0.14	0.56	0.40	0.41	0.42	0.37	0.62	0.42	0.42	0.39	0.12	0.36
3/B	1-(1.1′-Biphenyl-4-yl)ethan-1-one	n.a.	n.a.	0.21	n.a.	n.a.	n.a.	0.32	0.26	n.a.	0.22	0.30	0.35	0.36	0.28	n.a.	n.a.	0.14	0.15	n.a.	0.27
3/B	1-(4-Ethylphenyl)ethan-1-one	0.02	0.02	0.02	n.a.	n.a.	n.a.	0.04	0.02	0.03	0.03	0.03	0.05	0.05	n.a.	n.a.	n.a.	n.a.	n.a.	n.a.	0.04
3/B	2-(Methoxymethyl)-2-phenyl-1.3-dioxolane	n.a.	n.a.	n.a.	n.a.	n.a.	n.a.	n.a.	n.a.	n.a.	n.a.	n.a.	n.a.	n.a.	n.a.	n.a.	n.a.	n.a.	0.39	0.37	0.38
3/B	2.2′.4′.6′-Tetramethylpropiophenone	0.21	0.19	0.95	0.82	0.84	0.67	1.39	0.09	0.40	0.89	1.23	1.42	1.54	0.79	0.82	0.89	0.96	0.91	n.a.	1.42
3/B	4-(N-Methyl-N-methoxy)indane-1-carboxamide	0.11	n.a.	0.40	0.29	0.29	0.18	0.33	0.065	n.a.	0.31	0.44	0.41	0.32	n.a.	0.28	0.31	0.30	0.28	n.a.	0.38
3/B	Acetophenone	n.a.	n.a.	0.29	0.21	0.23	0.22	0.56	n.a.	0.13	0.37	0.40	0.75	0.61	0.23	0.24	0.24	0.29	0.27	n.a.	0.53
3/B	Fluoren-9-one	n.a.	n.a.	n.a.	n.a.	0.53	n.a.	0.33	n.a.	n.a.	0.50	0.40	n.a.	0.39	0.65	0.69	n.a.	n.a.	n.a.	n.a.	n.a.
3/C	4-Vinylbenzoic acid	0.68	0.71	1.30	0.86	0.84	0.32	0.25	0.62	0.62	0.98	0.35	0.41	0.81	0.82	1.03	0.60	0.84	0.79	0.26	0.79
3/C	trans-Vinyl cinnamate	0.52	0.63	1.42	1.20	1.29	1.15	1.74	0.58	0.62	1.33	1.86	1.79	1.84	1.22	1.24	1.34	1.47	1.50	n.a.	1.77
3/C	Vinyl benzoate	3.25	2.87	4.33	3.76	4.26	5.17	4.55	3.11	2.52	4.41	4.74	4.09	4.67	4.89	4.03	4.80	4.57	5.03	4.96	4.97
3/C	2.3-Dichlorophenyl 4-butylbenzoate	n.a.	n.a.	n.a.	n.a.	n.a.	n.a.	n.a.	n.a.	n.a.	0.15	0.23	0.14	0.19	n.a.	n.a.	n.a.	0.26	0.19	n.a.	0.21
3/C	2.5-Chlorophenyl 4-butylbenzoate	n.a.	n.a.	n.a.	n.a.	0.21	0.13	n.a.	n.a.	n.a.	n.a.	n.a.	n.a.	n.a.	n.a.	n.a.	0.22	n.a.	n.a.	n.a.	n.a.
3/C	4-Cyanophenyl 4-ethylbenzoate	0.42	0.39	1.20	0.92	1.00	0.52	0.82	0.62	0.48	0.74	0.91	0.93	0.89	0.25	1.06	0.93	1.07	1.06	0.36	0.85
3/C	4-Cyanophenyl meta-toluate	n.a.	n.a.	n.a.	n.a.	n.a.	n.a.	n.a.	n.a.	n.a.	n.a.	n.a.	n.a.	n.a.	0.46	n.a.	n.a.	n.a.	n.a.	n.a.	n.a.
3/C	4-Cyanophenyl 4-propylbenzoate	0.29	0.54	0.96	0.83	0.86	0.76	1.05	0.36	0.25	0.82	1.01	0.94	1.09	0.64	0.67	0.86	0.93	0.82	0.36	0.87
3/D	Bis(2-ethylhexyl) terephthalate (DEHT)	n.a.	n.a.	n.a.	n.a.	n.a.	n.a.	0.56	n.a.	2.74	n.a.	n.a.	n.a.	n.a.	n.a.	n.a.	n.a.	n.a.	n.a.	n.a.	n.a.
3/D	Bis(2-methylpropyl) phthalate	0.11	0.32	n.a.	n.a.	n.a.	n.a.	n.a.	n.a.	1.08	n.a.	n.a.	n.a.	n.a.	n.a.	n.a.	n.a.	n.a.	n.a.	n.a.	n.a.
3/D	Bis(2-ethylhexyl) adipate (DEHA)	n.a.	n.a.	0.15	0.15	n.a.	n.a.	n.a.	0.49	0.39	n.a.	n.a.	0.02	n.a.	n.a.	n.a.	n.a.	n.a.	n.a.	n.a.	n.a.
3/D	Diethyl phthalate (DEP)	n.a.	n.a.	9.50	n.a.	n.a.	n.a.	n.a.	n.a.	n.a.	4.31	1.61	0.02	n.a.	n.a.	n.a.	n.a.	n.a.	n.a.	1.71	n.a.
3/D	Cyclobutyl ethyl phthalate	n.a.	n.a.	n.a.	n.a.	n.a.	n.a.	n.a.	n.a.	n.a.	n.a.	n.a.	n.a.	n.a.	n.a.	n.a.	n.a.	n.a.	n.a.	n.a.	1.19
3/D	Diethylene glycol dibenzoate	0.32	0.28	0.32	0.22	0.27	0.25	0.19	0.24	0.20	0.35	0.42	0.39	0.25	0.25	0.26	0.31	0.31	0.32	0.16	0.18
3/D	Phenyl 4-butylbenzoate	n.a.	n.a.	0.22	0.21	n.a.	n.a.	n.a.	n.a.	n.a.	n.a.	n.a.	n.a.	n.a.	n.a.	0.19	n.a.	n.a.	n.a.	n.a.	n.a.
3/E	Triphenylphosphine oxide	n.a.	n.a.	n.a.	n.a.	n.a.	n.a.	n.a.	n.a.	n.a.	n.a.	n.a.	n.a.	n.a.	n.a.	n.a.	n.a.	n.a.	n.a.	0.38	n.a.
4/A	1.2′-Binaphthalene	n.a.	n.a.	n.a.	n.a.	n.a.	n.a.	n.a.	n.a.	n.a.	0.21	n.a.	n.a.	n.a.	n.a.	0.22	0.21	n.a.	n.a.	n.a.	n.a.
4/A	2.2′-Binaphthalene	0.19	n.a.	0.15	0.21	0.17	n.a.	n.a.	0.023	n.a.	0.10	n.a.	0.17	0.12	n.a.	0.20	0.25	0.24	n.a.	n.a.	n.a.
4/A	2.2′-Dimethyl-1.1′-biphenyl	n.a.	n.a.	0.08	0.06	0.04	n.a.	0.08	n.a.	n.a.	n.a.	0.01	n.a.	0.07	n.a.	n.a.	0.01	0.02	0.03	n.a.	0.01
4/B	3′-Methyl-1.1′:4′.1″-terphenyl	n.a.	n.a.	0.19	0.17	n.a.	n.a.	0.15	n.a.	n.a.	0.15	n.a.	0.17	n.a.	0.14	0.15	n.a.	0.20	n.a.	n.a.	n.a.
4/B	meta.meta-Quaterphenyl	n.a.	n.a.	0.14	0.19	n.a.	n.a.	0.16	n.a.	n.a.	0.19	0.15	0.21	0.17	n.a.	0.17	0.21	0.22	n.a.	n.a.	0.12
4/B	ortho-Terphenyl	0.16	n.a.	n.a.	n.a.	n.a.	0.20	n.a.	n.a.	n.a.	n.a.	n.a.	n.a.	n.a.	n.a.	n.a.	n.a.	n.a.	0.18	n.a.	n.a.
4/B	Triphenylene	0.26	0.36	0.62	0.56	0.49	0.55	0.27	n.a.	0.18	0.40	0.28	0.44	0.36	0.48	0.54	0.52	0.64	0.43	n.a.	0.25
4/C	8.9-Dihydro-7H-cyclopenta[a]pyrene	n.a.	n.a.	0.10	0.15	0.12	n.a.	n.a.	n.a.	n.a.	n.a.	n.a.	0.12	n.a.	n.a.	n.a.	n.a.	0.12	n.a.	n.a.	n.a.
4/C	9-Methylene-9H-fluorene	n.a.	n.a.	0.10	0.09	0.09	0.10	0.22	n.a.	n.a.	0.23	0.21	0.22	0.23	0.09	0.11	0.08	0.10	0.08	n.a.	0.18
4/C	(E.E)-1.4-Diphenyl-1.3-butadiene	n.a.	n.a.	n.a.	n.a.	n.a.	n.a.	n.a.	n.a.	n.a.	n.a.	n.a.	n.a.	n.a.	n.a.	n.a.	n.a.	n.a.	n.a.	0.16	n.a.
4/C	Naphthalene	n.a.	n.a.	0.14	0.05	0.12	0.05	n.a.	n.a.	n.a.	0.15	0.23	n.a.	0.36	0.04	0.05	0.06	0.12	0.05	n.a.	0.21
4/C	1-Phenylnaphthalene	n.a.	0.19	0.21	n.a.	0.18	0.25	0.22	0.18	n.a.	0.25	0.17	n.a.	0.26	0.23	n.a.	0.25	0.27	0.31	n.a.	n.a.
4/C	Phenanthrene	0.21	0.16	0.64	0.46	n.a.	0.71	n.a.	n.a.	n.a.	n.a.	n.a.	0.46	n.a.	n.a.	n.a.	0.51	0.68	0.57	n.a.	0.19
4/D	2-Methyl-3H-benz[e]indene	n.a.	n.a.	n.a.	0.15	n.a.	0.15	n.a.	n.a.	n.a.	n.a.	n.a.	n.a.	n.a.	n.a.	n.a.	0.17	0.11	n.a.	n.a.	n.a.
4/D	Benzene-tetrakis(cyclobutanyl)	n.a.	n.a.	n.a.	0.19	n.a.	n.a.	n.a.	n.a.	n.a.	n.a.	n.a.	n.a.	n.a.	n.a.	0.16	n.a.	n.a.	n.a.	n.a.	n.a.

Table S4. C-PET compound groups and their contribution to chemical load.

ID	Bottle name	Beverage	1/A Biphenyls and biphenyl-based derivatives		1/B Aromatic aldehydes derived from PET		1/D Terphenyls		1/E Benzoic acid and oxygenated benzoic derivatives	
			Account	Concentration (%)	Account	Concentration (%)	Account	Concentration (%)	Account	Concentration (%)
PET 1	Hanácká kyselka 1.5	FMW	3	0.46	1	0.19	1	0.19	4	13.86
PET 2	Hanácká kyselka 0.7	PW	3	0.44	1	0.21	1	0.21	4	14.93
PET3	Ondrášovka 1.5	FMW	2	0.28	2	0.24	1	0.32	4	14.97
PET 4	Ondrášovka 0.5	FMW	2	1.78	2	0.18	1	0.21	4	7.78
PET5	Mattoni	PW	3	3.17	2	0.36	1	0.61	4	18.79
PET6	Gemerka	FMW	3	5.33	2	0.33	1	0.52	4	18.86
PET7	Poděbradka Prolinie	FMW	3	3.19	2	0.3	1	0.52	4	13.31
PET8	Artesie	FMW	3	2.97	2	0.32	1	0.44	4	15.97
PET 9	Aquilla	PW	3	2.99	2	0.32	1	0.47	4	14.75
PET 10	Natura	PW	4	5.31	2	0.36	1	0.5	4	14.53
PET11	Magnesia Blue	PW	4	4.94	2	0.33	1	0.5	4	12.02
PET12	Vittel	PW	4	5.93	2	0.38	1	0.51	4	15.09
PET13	Evian	PW	4	4.92	2	0.39	1	0.43	4	14.29
PET14	Korunní	PW	5	6.59	2	0.44	1	0.12	4	20.37
PET15	Römerquelle	PW	1	0.51	1	0.08	0	0.01	4	1.68
PET17	Kofola	SD	5	7.77	2	0.49	1	0.73	4	21.56
PET19	Zonka	SD	5	4.68	2	0.61	1	0.32	4	9.13
PET20	Coca-Cola	SD	5	2.78	2	0.51	1	0.24	4	18.42
PET21	Magnesia Red	FMW	5	4.47	2	0.55	1	0.38	4	21.8
PET22	Kofola Targa	SD	5	4.15	2	0.55	1	0.15	4	4.88

ID	Bottle name	Beverage	1/F Terephthalic acid		1/G Secondary aromatic ketones		1/H Mixed origin: phenol and benzene		Total	
			Account	Concentration (%)	Account	Concentration (%)	Account	Concentration (%)	Account	Concentration (%)
PET 1	Hanácká kyselka 1.5	FMW	1	0.63	1	0.16	2	0.18	13	15.67
PET 2	Hanácká kyselka 0.7	PW	1	0.85	0	0	2	0.19	12	16.83
PET3	Ondrášovka 1.5	FMW	1	0.62	0	0.25	2	0.19	12	16.88
PET 4	Ondrášovka 0.5	FMW	1	0.4	1	0.58	1	0.19	12	11.12
PET5	Mattoni	PW	1	0.96	1	0.59	2	0.16	14	24.64
PET6	Gemerka	FMW	1	0.54	0	0.49	1	0.05	12	26.12
PET7	Poděbradka Prolinie	FMW	1	1.32	1	0.47	2	0.1	14	19.2
PET8	Artesie	FMW	1	0.5	1	0.56	2	0.18	14	20.95
PET 9	Aquilla	PW	1	0.63	1	0.5	2	0.23	14	19.9
PET 10	Natura	PW	1	0.82	1	0.6	1	0.36	14	22.49
PET11	Magnesia Blue	PW	1	0.67	1	0.46	1	0.18	14	19.1
PET12	Vittel	PW	1	0.76	1	0.56	2	0.18	15	23.41
PET13	Evian	PW	1	0.7	1	0.24	2	0.39	15	21.36
PET14	Korunní	PW	1	0.86	1	0.75	2	0.21	16	29.35
PET15	Römerquelle	PW	1	0.52	0	0	1	0.17	8	2.97
PET17	Kofola	SD	1	0.69	1	0.78	2	0.29	16	32.31
PET19	Zonka	SD	1	0.49	1	0.79	2	0.37	16	16.39
PET20	Coca-Cola	SD	1	0.74	1	0.76	1	0.15	15	23.6
PET21	Magnesia Red	FMW	1	0.41	1	0.72	2	0.42	16	28.75
PET22	Kofola Targa	SD	1	0.6	1	0	2	0.32	16	10.65

Explanations: FMW = Flavoured mineral water; PW = Plain/mineral water; SD = Soft drink/soda

Table S5. Distribution of externally derived compounds (E-PS/PVC/FCM), including group composition, compound counts and relative abundances across PET bottle types.

Number	Bottle name	Beverage	3/A Low-molecular-weight aromatic hydrocarbons		3/B Aromatic ketones and related carbonyl compounds		3/C Benzoate-type esters		Total		Plasticisers occurrence
			Account	Concentration %	Account	Concentration %	Account	Concentration %	Account	Concentration %	
PET 1	Hanácká kyselka 1.5	FMW	5	0.59	3	0.34	6	5.48	14	6.4	Bis (2-methylpropyl)phthalate
PET 2	Hanácká kyselka 0.7	PW	4	0.59	2	0.21	6	5.42	12	6.22	Bis (2-methylpropyl)phthalate
PET5	Mattoni	PW	9	1.15	5	1.86	6	9.52	20	12.53	DEP. DEHA
PET7	Poděbradka Prolinie	FMW	6	0.87	3	1.32	6	7.8	15	9.98	DEHA
PET 9	Aquilla	PW	8	1.01	4	1.88	6	8.51	18	11.4	Not detected
PET11	Magnesia Blue	PW	5	0.99	3	1.06	6	8.17	14	10.23	Not detected
PET21	Magnesia Red	FMW	9	1.48	6	2.96	6	8.61	21	13.05	DEHT
PET3	Ondrášovka 1.5	FMW	8	1.02	4	0.43	6	5.53	18	6.98	DEHT.DEHA
PET 4	Ondrášovka 0.5	FMW	5	0.43	3	0.57	6	4.69	14	5.68	Bis (2-methylpropyl)phthalate
PET14	Korunní	PW	8	1.38	6	2.32	7	8.77	2	12.47	DEP
PET17	Kofola	SD	9	1.41	6	2.8	7	9.51	22	13.72	DEP
PET19	Zonka	SD	8	1.53	5	2.98	7	8.69	20	13.21	DEP
PET22	Kofola Targa	SD	8	1.44	6	3.27	7	9.74	21	14.46	Not detect
PET6	Gemerka	FMW	5	0.91	4	1.95	6	8.07	15	10.94	Not detected
PET8	Artesie	FMW	8	1.33	4	2.02	6	8.28	18	11.63	Not detected
PET 10	Natura	PW	5	1.08	3	1.44	6	8.84	14	11.37	Not detected
PET12	Vittel	PW	6	1.17	4	1.69	7	9.45	17	12.30	Not detected
PET13	Evian	PW	8	1.15	4	1.6	7	9.7	19	12.46	Not detected
PET15	Römerquelle	PW	2	0.14	0	0	6	6.10	8	6.24	DEP
PET20	Coca-Cola	SD	7	1.39	5	2.64	7	9.65	19	13.68	Not detected

Explanations: FMW = Flavoured mineral water; PW = Plain/mineral water; SD = Soft drink/soda.

Table S6. Occurrence and relative contribution of condensed aromatic structures (A-AROM) in PET bottles.

Number	Bottle name	Beverage	4/A Biphenyl-type recombination products		4/B Higher-order condensed aromatics		4/C Polycyclic aromatic structures		Total	
			Account	Concentration %	Account	Concentration %	Account	Concentration %	Account	Concentration %
PET 1	Hanácká kyselka 1.5	FMW	1	0.19	1	0.26	1	0.21	3	0.66
PET 2	Hanácká kyselka 0.7	PW	0	0	1	0.36	2	0.35	3	0.71
PET 3	Ondrášovka 1.5	FMW	1	0.02	0	0	1	0.18	2	0.2
PET 4	Ondrášovka 0.5	FMW	0	0	1	0.18	0	0	1	0.18
PET 5	Mattoni	PW	2	0.23	3	0.95	5	1.19	10	2.39
PET 6	Gemerka	FMW	0	0	2	0.62	3	0.35	5	0.97
PET 7	Poděbradka Prolinie	FMW	2	0.27	3	0.91	4	0.76	9	1.94
PET 8	Artesie	FMW	1	0.2	3	0.87	2	0.16	6	1.22
PET 9	Aquila	PW	2	0.2	1	0.49	4	0.5	7	1.19
PET 10	Natura	PW	2	0.26	2	0.73	4	0.89	8	1.88
PET 11	Magnesia Blue	PW	0	0	1	0.55	4	1.11	5	1.66
PET 12	Vittel	PW	2	0.26	3	1.6	5	1.29	10	2.6
PET 13	Evian	PW	1	0.03	1	0.43	4	1	6	1.46
PET 14	Korunní	PW	1	0.1	3	0.73	3	0.63	7	1.46
PET 15	Römerquelle	PW	0	0	0	0	0	0	0	0
PET 17	Kofola	SD	1	0.01	2	0.43	3	0.6	6	1.5
PET 19	Zonka	SD	1	0.17	3	0.83	3	0.8	7	1.8
PET 20	Coca-Cola	SD	1	0.01	2	0.38	3	0.59	6	0.98
PET 21	Magnesia Red	FMW	1	0.08	3	0.59	2	0.44	6	1.11
PET 22	Kofola Targa	SD	2	0.19	2	0.53	3	0.85	7	1.58

Explanations: FMW = Flavoured mineral water; PW = Plain water; SD = Soft drink. Relative abundances are expressed as percentage peak areas.
